# Accumulation of microbial DNAs promotes to islet inflammation and β cell abnormalities in obesity in mice

**DOI:** 10.1038/s41467-022-28239-2

**Published:** 2022-01-28

**Authors:** Hong Gao, Zhenlong Luo, Yudong Ji, Kechun Tang, Zhongmou Jin, Crystal Ly, Dorothy D. Sears, Sushil Mahata, Wei Ying

**Affiliations:** 1 Division of Endocrinology & Metabolism, University of California, San Diego, La Jolla, CA 92093 USA; 2 Department of Gastroenterology, Tongji Hospital, Tongji medical College, Huazhong University of Science and Technology, 430030 Wuhan, China; 3 Department of Anesthesiology, Institute of Anesthesiology and Critical Care, Union Hospital, Tongji medical College, Huazhong University of Science and Technology, 430022 Wuhan, China; 4 VA San Diego Healthcare System, La Jolla, CA 92093 USA; 5 Division of Biological Sciences, University of California, San Diego, La Jolla, CA 92093 USA; 6 College of Health Solutions, Arizona State University, Phoenix, AZ 85004 USA; 7 Department of Medicine, University of California, San Diego, La Jolla, CA 92093 USA; 8 Department of Family Medicine, University of California, San Diego, La Jolla, CA 92093 USA; 9 Moores Cancer Center, University of California, San Diego, La Jolla, 92093 CA USA

**Keywords:** Monocytes and macrophages, Diabetes, Metabolic diseases

## Abstract

Various microbial products leaked from gut lumen exacerbate tissue inflammation and metabolic disorders in obesity. Vsig4+ macrophages are key players preventing infiltration of bacteria and their products into host tissues. However, roles of islet Vsig4+ macrophages in the communication between microbiota and β cells in pathogenesis of obesity-associated islet abnormalities are unknown. Here, we find that bacterial DNAs are enriched in β cells of individuals with obesity. Intestinal microbial DNA-containing extracellular vesicles (mEVs) readily pass through obese gut barrier and deliver microbial DNAs into β cells, resulting in elevated inflammation and impaired insulin secretion by triggering cGAS/STING activation. Vsig4+ macrophages prevent mEV infiltration into β cells through a C3-dependent opsonization, whereas loss of Vsig4 leads to microbial DNA enrichment in β cells after mEV treatment. Removal of microbial DNAs blunts mEV effects. Loss of Vsig4+ macrophages leads to microbial DNA accumulation in β cells and subsequently obesity-associated islet abnormalities.

## Abstract (web summary)

Obesity is associated with increased gut permeability, and microbial products that are leaked from the gut may contribute towards obesity-associated inflammation. Here the authors show that the leakage of gut extracellular vesicles containing microbial DNA leads to bacterial DNA accumulation in pancreatic β-cells, promoting obesity-associated islet inflammation.

## Introduction

As a hallmark of obesity, low-grade tissue inflammation manifests in various organs, including adipose tissue, liver, muscle, and pancreatic islets^1,2^. Chronic islet inflammation exists in a variety of mouse models of obesity and type 2 diabetes mellitus (T2DM) as well as in the islets from patients with obesity/T2DM^3–6^. This obesity-induced islet inflammation contributes to β cell abnormalities that characterize T2DM. However, the mechanism underlying obesity-induced islet inflammation is not fully understood.

Healthy subjects are characterized by a diverse composition of the intestinal microbiota and a normal functional intestinal barrier^7^. By contrast, obesity exhibits profound functional and compositional alternations in gut microbiota, collectively termed dysbiosis^8^. Additionally, gut barrier breach is a well-recognized feature of obesity, resulting in the leakage of microbiota-derived products into the circulation and distant organs of host^7,9^. More importantly, emerging evidence indicates microbial metabolites can exacerbate tissue inflammation and metabolic disorders in the context of obesity^10–12^. Previous studies suggest that microbiota-derived products could be leaked into islets and contribute to the development of T1DM^13^. Recent studies have also shown that obesity is accompanied by the enrichment of microbial DNAs in the circulation and metabolic tissues of both humans and mice^14–17^. In addition, circulating microbial DNAs may be signatures predicting the development of metabolic diseases^15,18^. There is clear evidence that extracellular vesicles (EVs) can transport various cargos, including RNAs, DNAs, proteins, and lipids, from one cell type to other neighbor or distant cells^19^. Emerging evidence has revealed that the EV-mediated cellular communications exert profound regulation on obesity-associated metabolic responses^20–23^. A wide range of microbiota species can produce EVs which can be released into host circulation^24,25^. Thus, this leads us to hypothesize that microbiota-derived EVs encapsulating microbial DNAs (mEVs) play a role in mediating islet inflammation and β cell dysfunction in the context of obesity.

Macrophages are the dominant immune cell type residing in islets^6^. Several studies have observed a significant increase in islet macrophage population in both patients with obesity and obese animal models, compared to lean individuals^3,26,27^. Our recent study has also revealed that the expansion and functional switch of islet macrophages contribute to the development of islet inflammation which mediates β cell proliferation and insulin secretion in the context of obesity^3^. In the normal, lean state, islet macrophages are the key players in balancing islet inflammatory microenvironment^6^. For example, the complement receptor of immunoglobulin family (Vsig4+) islet macrophages, a group of innate immune cells of the complement system for phagocytosis of the circulating pathogens, can establish a protective barrier surrounding pancreatic islets to prevent the incidence of autoimmune responses and T1DM^28,29^. In addition, Vsig4+ Kupffer cells in the liver can efficiently clear bacteria and their products from the portal vein draining the intestine^17,30,31^. The ability of Vsig4+ macrophages to capture foreign materials mainly depends on complement protein C3-mediated opsonization^17,30^. However, the roles of islet Vsig4+ macrophages in preventing the development of obesity-associated islet inflammation and β cell abnormalities are still unknown.

Here, we show that intestinal mEVs can be translocated into host circulation and transfer microbial DNAs into pancreatic β cells in the context of obesity, resulting in increased islet inflammation and β cell abnormalities. Islet Vsig4+ macrophages can block the infiltration of intestinal mEVs into β cells through a C3-mediated mechanism, whereas prolonged obesity causes a remarkable decrease in Vsig4+ macrophage population, allowing the spread of intestinal mEVs and microbial DNA enrichment within β cells of both obese mice and patients with obesity. Depletion of microbial DNAs blunts the pathogenic effects of intestinal mEVs. Accumulation of bacterial DNAs can initiate inflammatory response and impair insulin secretion of β cells through triggering the activation of cGAS/STING pathway.

## Results

### Microbial DNA-containing extracellular vesicles are translocated from gut into pancreatic β cells of obese host

**Fig. 1:**
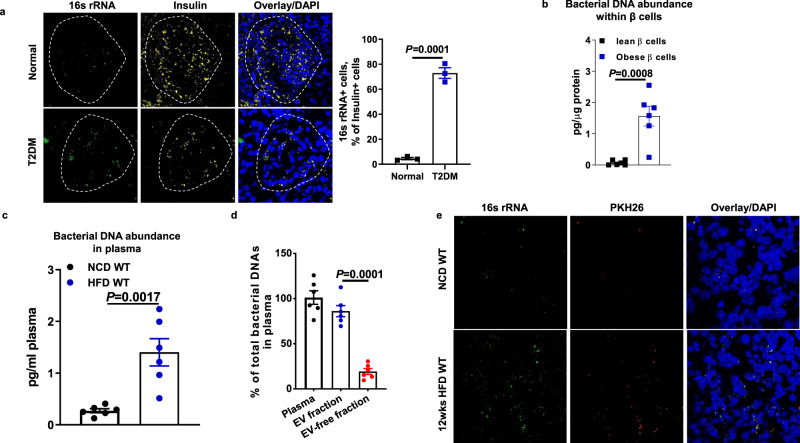
Obesity induces the leakage of microbial DNA containing extracellular vesicles (mEVs) from the gut lumen into pancreatic β cells. **a** Bacterial DNA abundance within the insulin+ cells in the pancreas of both healthy humans and patients with type 2 diabetes (T2DM). Left, representative images; right, statistics of the number of 16s rRNA+insulin+ cells per islet and each dot represents the mean value from each donor. *n* = 3 normal heathy donors and *n* = 3 T2DM donors. Scale bar = 30 µm. **b** 16 s rRNA levels within the GFP + β cells sorted from lean or 12 weeks high-fat diet (12wks HFD)-fed MIPGFP mice (*n* = 6 per group). **c** Bacterial DNA abundance in the circulation of both NCD WT and 12wks HFD WT mice (*n* = 6 per group). **d** The abundance of bacterial DNAs in the EVs or EV-free fraction of 12wks HFD mouse plasma (*n* = 6 per group). **e** The intensities of 16 s rRNA and PKH26 fluorescence within the pancreas of both NCD WT and 12wks HFD recipient mice after 24 h of intravenous injection with PKH26-labeled intestinal EVs. Scale bar = 30 µm. Representative images are from three independent experiments. All experiments were repeated at least twice with similar results. Data are presented as the mean ± SEM. *P* values are determined by unpaired two-sided Student’s *t* test (**a**–**d**).

We first examined the abundance of bacterial DNAs within the pancreas of either healthy individuals or patients with obesity/T2DM with 16s rRNA probes. As shown in Fig. 1a, bacterial DNAs were markedly enriched in pancreatic β cells of patients with obesity/T2DM, as evidenced by a robust 16s rRNA intensity within insulin+ cells. By contrast, healthy human islets barely contained bacterial DNAs. In addition, we validated a minimal level of external bacterial DNA contamination during these tests, as demonstrated by no 16s rRNA signal detected in the pancreas of 12 weeks high-fat diet feeding (12wks HFD) germ-free WT mice (Fig. S1a). qPCR analysis also indicates that GFP + β cells isolated from 12wks HFD MIPGFP mice contained high levels of 16s rRNAs, whereas bacterial DNAs were rarely detectable within the β cells from normal chow diet (NCD) MIPGFP mice (Fig. 1b). Taken together, these results indicate that bacterial DNAs are significantly accumulated in the pancreatic β cells of obese host.

Consistently, more bacterial DNAs were found in the bloodstream of 12wks HFD mice, compared to NCD mice (Fig. 1c). More importantly, most of circulating bacterial DNAs were bound with EVs, as evidenced by much more 16s rRNAs within plasma EVs than in the non-EV plasma fraction (Fig. 1d). Similarly, bacterial DNAs were enriched in plasma from individuals with obesity compared to plasma from lean participants, in which the bacterial DNA concentration was extremely low (Fig. S1b). Thus, we next tested whether gut mEVs can be translocated from gut lumen into host pancreatic islets. EVs were isolated from the small intestinal lumen contents of 16wks HFD fed WT mice, and qPCR analysis confirms that these gut EVs harbored microbial DNAs (Figs. S1c–e). In addition, other components such as the levels of EV markers, fatty acids, RNAs were not significantly different between lean and obese gut mEVs (Figs. S1f–h). To further examine if intestinal EVs pass through gut barrier, intestinal EVs were conjugated with PKH26 red fluorescent dye and injected (5 × 10^9^ EVs per mouse) into the jejunum section of either NCD or 12wks HFD WT mice. After 24 h, robust red fluorescent signals were presented in the pancreas of obese recipient mice, whereas much less amount of PKH26 EVs was leaked into NCD pancreatic tissues (Figs. 1e and S1i). In addition, the majority of PKH26 EVs transferred microbial DNAs into the pancreas of obese recipients, as shown by co-localization of most 16s rRNA and PKH26 signals (Fig. 1e). Thus, these results suggest that microbial DNA-containing EVs can pass through gut barrier and reach host pancreas in the context of obesity.

### Vsig4+ macrophages can prevent islets from the infiltration of intestinal mEVs

**Fig. 2:**
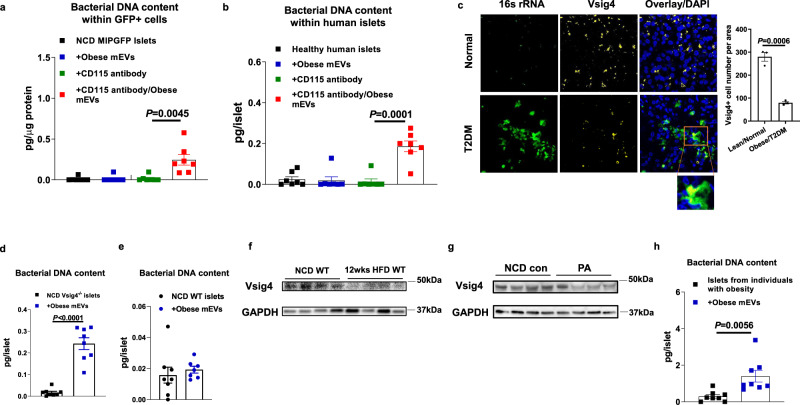
Vsig4+ macrophages block the infiltration of obese mEVs into pancreatic β cells. Obese mEVs-mediated bacterial DNA accumulation within mouse GFP + β cells (**a**, *n* = 7 per group) or healthy human islets (**b**, *n* = 7 per group) with CD115 antibody-induced macrophage depletion. **c** The abundance of 16 s rRNA and Vsig4 within the pancreas of both healthy humans and patients with T2DM. Scale bar = 30 µm. Representative images are from *n* = 3 normal healthy donors and *n* = 3 T2DM donors. After in vitro treatment with obese mEVs, bacterial DNA levels within either NCD Vsig4^−/−^ (**d**, *n* = 8 per group) or NCD WT islets (**e**, *n* = 7 per group). Vsig4 abundance within pancreas after 12wks HFD feeding (**f**) or 2wks of palmitate acid treatment (**g**). **h** Bacterial DNA abundance within obese mEVs-treated islets derived from patients with obesity (*n* = 8 per group). All experiments were repeated at least twice with similar results. Data are presented as the mean ± SEM. *P* values are determined by unpaired two-sided Student’s *t* test (**a**–**e**, **h**). Source data are provided as a Source data file.

Tissue-resident macrophages exert profound protection blocking the invasion of bacteria and their byproducts. We found that these islet macrophages can prevent the infiltration of mEVs, as evidenced by very low/non-detectable levels of bacterial DNAs in GFP + β cells after NCD MIPGFP WT islets treated with obese mEVs (Fig. 2a). By contrast, as a result of CD115 antibody-mediated depletion of islet macrophages, the abundance of bacterial DNAs was significantly increased in mouse GFP + β cells after NCD islets treated with obese mEVs (Figs. 2a and S2a). Similarly, we also observed that bacterial DNAs were significantly enriched in macrophage-depleted healthy human islets after treatment with obese mEVs, whereas bacterial DNA accumulation did not occur in healthy human islets treated with obese mEVs (Fig. 2b).

Previous studies have shown that Vsig4 plays a critical role in the interaction between tissue-resident macrophages and foreign particles^30^. Consistently, the majority of 16s rRNAs were co-localized with Vsig4+ macrophages in both healthy human and NCD WT mouse pancreas (Figs. 2c and S2b). We observed that there were barely detectable bacterial DNAs within the islets and bloodstream of both lean Vsig4^−/−^ and WT mice (Fig. S2c). Additionally, we found that microbial DNAs were readily accumulated within NCD Vsig4^−/−^ islets treated with obese mEVs, whereas there was much lower/non-detectable level of 16s rRNAs within NCD WT islets after treatment with obese mEVs (Fig. 2d, e). We also observed that obese mEVs accumulated in pancreatic α cells of lean Vsig4^−/−^ mice intravenously injected with PKH26-labeled mEVs (Fig. S2d). Consistent with previous studies, we found that obese mEVs infiltrated into the liver and heart of lean Vsig4^−/−^ mice, concomitant with elevated bacterial DNA abundance (Fig. S2e). Interestingly, compared to lean islets, there was a marked reduction in the population of Vsig4+ cells in the islets of 12wks HFD WT mice and patients with T2DM/obesity (Figs. 2c, f and S2f). In addition, after 2 weeks of oral gavage of palmitate acid/corn oil mixture into NCD WT mice, the abundance of Vsig4 in islets was significantly reduced, compared to control NCD mice without lipid treatment (Fig. 2g). Microbiota and their byproducts had minimal effect on Vsig4 expression, as evidenced by a significant reduction in Vsig4 abundance in the islets of 12wks HFD germ-free WT mice compared to NCD germ-free WT mice (Fig. S2g). Obese mEV treatment also did not significantly affect Vsig4 expression in the islets of NCD WT recipient mice (Fig. S2h). Concomitant with obesity-associated reduction in Vsig4 abundance, bacterial DNAs were significantly accumulated in both patients with obesity and obese mouse islets after treatment of obese mEVs (Figs. 2h and S2i). Thus, these data suggest the important role of Vsig4+ macrophages in preventing islets from the penetration of mEVs.

### Microbiota-derived EVs cause obesity-associated islet inflammation and β cell abnormalities

**Fig. 3:**
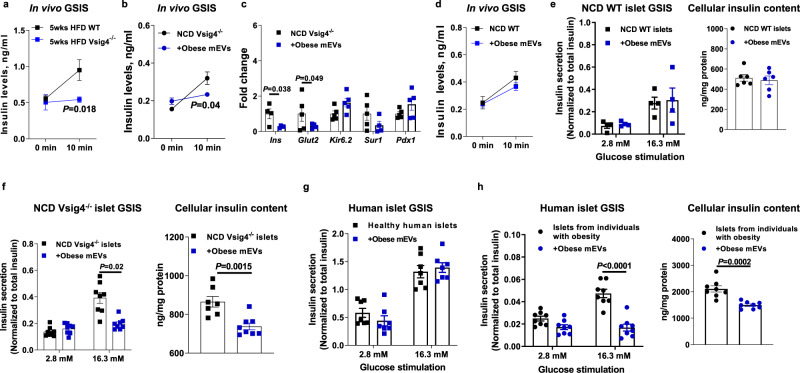
Obese mEVs cause obesity-associated islet inflammation and β cell abnormalities. **a** The glucose-stimulated insulin secretion (GSIS) levels of 5wks HFD WT and Vsig4^−/−^ mice (*n* = 5 per group). Effects of obese mEV treatment on GSIS (**b**, *n* = 5 per group) and the expression of key genes associated with β cell functions (**c**, *n* = 4–5 per group) in NCD Vsig4^−/−^ recipient mice. **d** The GSIS levels of NCD WT recipient mice after 4 weeks of obese mEV treatment (*n* = 7 per group). The GSIS and cellular insulin levels of either NCD WT (**e**, *n* = 4–6 per group) or NCD Vsig4^−/−^ (**f**, *n* = 7-8 per group) islets after in vitro treatment with obese mEVs. Effects of obese mEVs on GSIS and cellular insulin content of healthy human islets (**g**, *n* = 8 per group) or islets derived from patients with obesity (**h**, *n* = 8 per group). All experiments were repeated at least twice with similar results. Data are presented as the mean ± SEM. *P* values are determined by unpaired two-sided Student’s *t* test (**a**–**h**).

Given that microbiota-derived products can exacerbate tissue inflammation and metabolic disorders in obesity, we next assessed whether the accumulation of bacterial DNAs can trigger islet inflammation and β cell dysfunction. We found that 5wks HFD Vsig4^−/−^ mice exhibited a greater level of bacterial DNAs in islets compared to 5wks HFD WT mice, concomitant with increased proinflammatory cytokine *Il1b* abundance in these islets (Fig. S3a, b). In addition, 5wks HFD Vsig4^−/−^ mice produced lower insulin levels than 5wks HFD WT mice after glucose injection (Fig. 3a). We also observed that there was comparable abundance of *Ins, Glut2, Pdx1, and Sur1* in the islets between lean Vsig4^−/−^ and WT mice (Fig. S3c).

We next examined the effects of obese mEVs on triggering islet inflammation and β cell abnormalities. To mimic the enrichment of gut mEVs in bloodstream in obesity, either NCD WT or Vsig4^−/−^ recipient mice were treated with these mEVs through tail vein injection (5 × 10^9^ EVs per mouse), and mice were treated with mEVs twice per week to maintain enough mEVs accumulated in islets (Fig. S3d). While all recipient mice had similar body weight after 4 weeks mEV treatment, obese mEV treatment led to similar levels of bacterial DNAs in islets with that in 12wks HFD WT mice (Fig. S3d). More importantly, treatment with obese mEVs elevated islet inflammation in NCD Vsig4^−/−^ recipient mice, as evidenced by increased abundance of CD11c + macrophages and IL1β in islets (Figs. S3e and S3f). Additionally, NCD Vsig4^−/−^ recipient mice treated with obese mEVs had significantly lower levels of insulin secretion in response to glucose injection and impaired glucose tolerance (Figs. 3b and S3g). Consistently, the expression of *Ins* and *Glut2* associated with insulin production and secretion was downregulated in the NCD Vsig4^−/−^ mice treated with obese mEVs (Fig. 3c). By contrast, after 4 weeks obese mEV treatment, NCD WT recipient mice had similar islet responses and expression levels of key genes associated with insulin secretion and production with the control NCD WT mice without mEV injection (Figs. 3d and S3h). Consistently, the results from ex vivo mouse islet experiments show that obese mEV treatment had minimal effect on the glucose-stimulated insulin secretion (GSIS) and cellular insulin content of NCD WT islets (Fig. 3e), whereas NCD Vsig4^−/−^ islets exhibited impaired insulin production and insulin secretion in response to glucose stimulation after obese mEV treatment (Figs. 3f and S3i). In addition, to evaluate the impact of gut mEVs on metabolic phenotypes in the presence of other microbiota-derived products, lean Vsig4^−/−^ mice were treated with the combination of liposaccharide (LPS) and gut mEVs. Control Vsig4^−/−^ mice were only treated with LPS. After 4wks treatment, we observed that obese mEVs further promoted islet dysfunctions in lean Vsig4^−/−^ mice in the present of liposaccharide (LPS) (Figs. S3j–l). We also confirmed that healthy human islets had a non-significant change in GSIS in the present of obese mEVs (Fig. 3g). By contrast, as a result of decreased Vsig4 expression, islets from individuals with obesity exhibited worse GSIS and cellular insulin content after treatment with obese mEVs (Figs. 3h and S3m). Thus, obese mEVs can trigger islet inflammation and β cell dysfunction in the context of Vsig4+ macrophage absence.

**Fig. 4:**
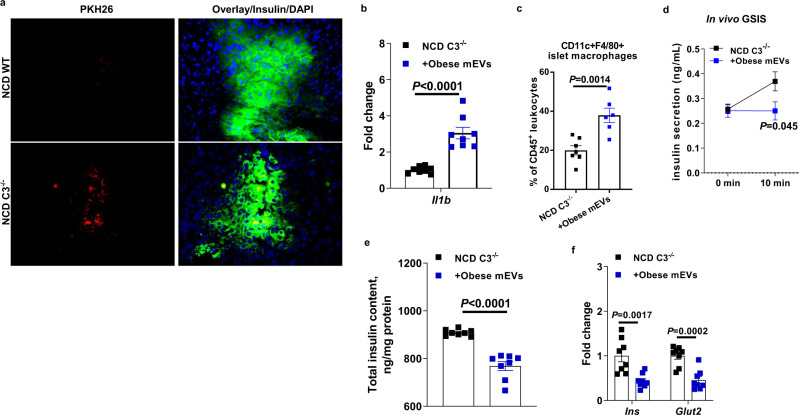
Complement component 3 (C3) facilitates the capture of mEVs by islet Vsig4+ macrophages. **a** The intensity of PKH26 fluorescence within islets of NCD C3^−/−^ mice after 24 h of intravenous injection with PKH26-labeled obese mEVs. Scale bar = 20 µm. Representative images are from three independent experiments. Effects of obese mEV treatment on islet *Il1b* abundance (**b**, *n* = 8 per group), islet macrophage population (**c**, *n* = 6 per group), GSIS (**d**, *n* = 8 per group), and insulin abundance (**e**, *n* = 8 per group) and the expression of key genes associated with insulin synthesis and secretion (**f**, *n* = 8 per group) in NCD C3^−/−^ mice. All experiments were repeated at least twice with similar results. Data are presented as the mean ± SEM. *P* values are determined by unpaired two-sided Student’s *t* test (**b**–**f**).

Vsig4+ macrophages rely on complement component C3-mediated opsonization to recognize microbial products^30^. Thus, we next evaluated the importance of C3 protein on mediating the interaction between Vsig4+ macrophages and mEVs. While obesity induced different patterns of C3 expression in β cells and islet macrophages, we observed no significant difference in pancreatic C3 abundance between lean and obese mice (Fig. S4a). We observed no difference in the abundance of key genes associated with insulin secretion and production between lean WT and C3^−/−^ mice (Fig. S4b). In line with previous findings^30^, C3 depletion impaired the ability of Vsig4+ macrophages to block gut mEVs from bloodstream, as evidenced by a robust PKH26 intensity in the insulin+ cells of NCD C3^−/−^ mice after intravenous injection of PKH26 mEVs (Fig. 4a). As a result of penetration of mEVs into β cells, NCD C3^−/−^ mice treated with obese mEVs had remarkably elevated inflammatory responses in islets (Fig. 4b, c), concomitant with decreased levels of GSIS, cellular insulin content, and glucose tolerance (Figs. 4d–f and S4c). Thus, these data suggest the critical role of C3 in the ability of Vsig4+ macrophages to clear mEVs.

A previous study reports that Vsig4+ macrophages interact with lipoteichoic acid (LTA) component of bacteria^31^. However, LTA was undetected from obese mEVs (Fig. S4d). In addition, we assessed the possibility that Vsig4+ macrophages capture mEVs through the LTA-mediated mechanism. Lean mice were intravenously injected with purified LTA (1 mg/mouse) to sufficiently bind to Vsig4+ macrophages. After 12 h LTA treatment, PKH26-labeled obese mEVs were intravenously injected into these lean mice. However, we did not detect PKH26 red fluorescent signal in the islets of these LTA-treated mice (Fig. S4e), suggesting the minimal effect of LTA on the sequestration of Vsig4+ macrophages on gut mEVs.

### Islet Vsig4+ macrophages are sufficient to block the pathogenic effects of gut mEV

**Fig. 5:**
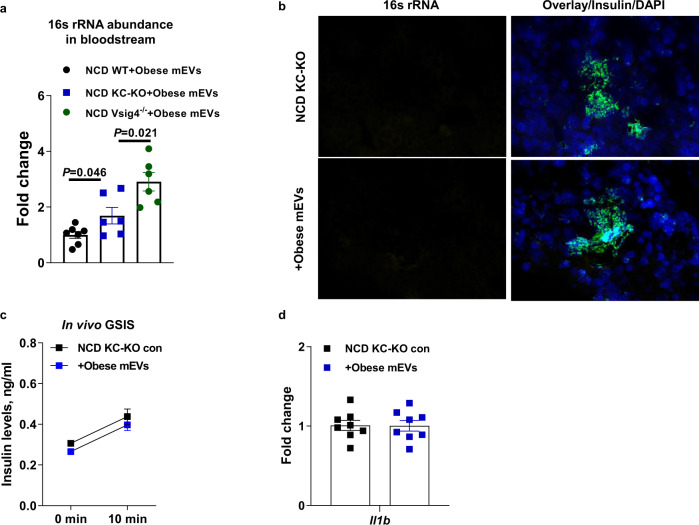
Islet Vsig4+ macrophages exert profound protection on β cells from the pathogenesis of gut mEVs. **a** qPCR analysis of 16 s rRNA abundance in plasma of lean WT, diphtheria toxin-treated Clec4fCre+DTR + (KC-KO), or Vsig4^−/−^ mice after 24 h intravenously injected with obese mEVs (*n* = 6 per group). **b** 16 s rRNA abundance in the pancreas of lean KC-KO mice after 4 weeks treatment with obese mEVs. Scale bar = 20 µm. Representative images are from three independent experiments. Effect of obese mEVs on the GSIS (**c**, *n* = 8 of KC-KO con, *n* = 6 of KC-KO + Obese mEVs) and islet *Il1b* abundance (**d**, *n* = 8 per group) in lean KC-KO mice. All experiments were repeated at least twice with similar results. Data are presented as the mean ± SEM. *P* values are determined by unpaired two-sided Student’s *t* test (**a**).

Previous studies have shown that the liver harbors high abundance of Vsig4+ macrophages and plays a crucial role in filtering gut mEVs and other pathogens. We next assessed whether islet Vsig4+ macrophages, in the absence of liver Vsig4+ macrophages, efficiently protect islet from the pathogenic impacts of gut mEVs. Given that Vsig4+ cells are Kupffer cells in the liver, hepatic Vsig4+ cells were depleted in lean Clec4fCre+DTR + mice treated with diphtheria toxin (DT) (KC-KO mice; Fig. S5a). In addition, islet Vsig4 abundance of lean Clec4fCre+DTR + mice was not affected after DT treatment (Fig. S5b). We observed that the levels of bacterial DNAs in the bloodstream of obese mEVs-treated lean KC-KO mice were less than that in lean Vsig4^−/−^ treated with obese mEVs (Fig. 5a). In addition, obese mEVs treatment led to greater levels of circulating bacterial DNAs in KC-KO compared to obese mEVs-treated lean WT mice (Fig. 5a). More importantly, after 4wks treatment with obese mEVs into lean KC-KO mice, 16s rRNA was undetected within islets, thus demonstrating that islet Vsig4+ macrophages can efficiently prevent the infiltration of gut mEVs (Fig. 5b). Consistently, we observed that obese mEV treatment had minimal effects on the metabolic phenotypes and islet inflammation of lean KC-KO mice (Figs. 5c, d and S5c–e). Thus, these data indicate that islet macrophages exert profound protection from the effects of gut mEVs.

### Microbial DNAs are pathogenic cargos contributing to the effects of gut EVs

**Fig. 6:**
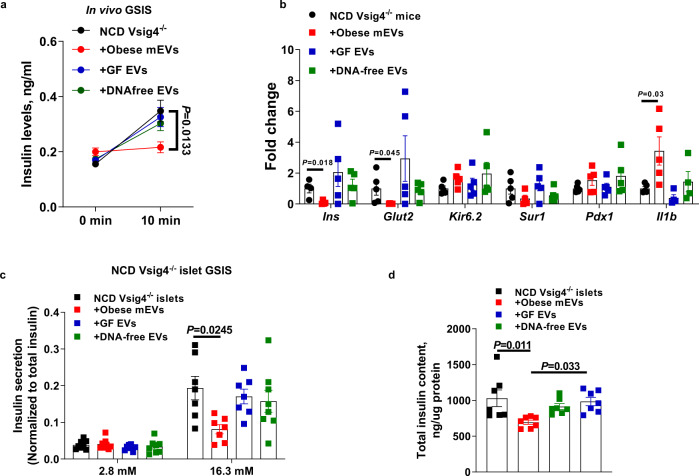
Pathogenic effects of microbiota-derived EVs on inducing obesity-associated islet inflammation and β cell abnormalities. Effects of intestinal EVs on the GSIS (**a**, *n* = 5 per group) and the abundance of key genes related to β cell functions (**b**, *n* = 5 per group) in NCD Vsig4^−/−^ mice. The GSIS levels (**c**, *n* = 7 per group) and cellular insulin content (**d**, *n* = 7 per group) of NCD Vsig4^−/−^ islets after in vitro treatment with obese mEVs, germ-free intestinal EVs (GF EVs), or DNA-free intestinal EVs. All experiments were repeated at least twice with similar results. Data are presented as the mean ± SEM. *P* values are determined by one-way ANOVA analysis (**a**–**d**).

We further validated whether microbiota-derived EVs among these intestinal EVs result in islet inflammation and β cell abnormalities. To address this, intestinal EVs were collected from the small intestinal contents of 12wks HFD germ-free mice (GF EVs; Fig. S6a) and then delivered into NCD Vsig4^−/−^ mice through tail vein injection (5 × 10^9^ EVs per mouse, twice per week). Another group of NCD Vsig4^−/−^ mice were injected with obese mEVs. As expected, obese mEV treatment blunted GSIS in NCD Vsig4^−/−^ recipient mice (Fig. 6a). However, GF EVs did not significantly change the β cell responses of NCD Vsig4^−/−^ recipient mice, thus demonstrating the pathogenesis of microbiota-derived EVs on islet function (Fig. 6a).

Given that microbial DNAs are important cargos with intestinal EVs, we also tested the contribution of microbial DNAs to the effects of these gut EVs. To generate microbial DNA-free EVs, obese gut EVs were treated with DNase after electroporation (Fig. S6b). Notably, treatment of microbial DNA-free EVs did not significantly affect the islet function of NCD Vsig4^−/−^ recipient mice (Figs. 6a and S6c). In addition, in vitro treatment of either GF EVs or DNA-free EVs had minimal effect on insulin secretion and cellular insulin content of the islets isolated from NCD Vsig4^−/−^ mice (Fig. 6b–d). Min6 cells treated with obese mEVs displayed decreased levels of GSIS and intracellular insulin content, whereas GF EV or DNA-free EV treatment had non-significant impact on Min6 insulin secretion (Fig. S6d–f). Overall, these results demonstrate that microbial DNAs are the key pathogenic cargos within obese gut EVs that can induce islet dysfunction.

### Microbial DNAs promote the activation of cGAS/STING pathway that triggers islet inflammation

**Fig. 7:**
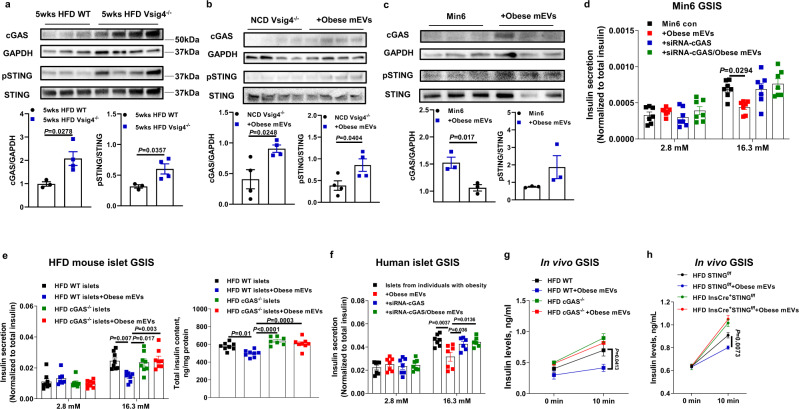
The activation of cGAS/STING pathway plays a critical role in obese mEV-induced islet inflammation. **a** The abundance of cGAS/STING in the islets of 5wks HFD WT and Vsig4^−/−^ mice. **b** The activation of cGAS/STING pathway in the islets of NCD Vsig4^−/−^ recipient mice after 4 weeks of obese mEV treatment. **c** Effect of obese mEV treatment on cGAS/STING abundance within Min6 cells. The importance of cGAS on the ability of obese mEVs to regulate the GSIS of Min6 cells (**d**, *n* = 7 per group), obese islets (**e**, *n* = 8 per group), or islets from individuals with obesity (**f**, *n* = 7 per group). **g** The GSIS levels of 8wks HFD cGAS^−/−^ or WT mice injected with obese mEVs (*n* = 5 per group). **h** The GSIS levels of 8wks HFD STING^f/f^ or InsCre^+^STING^f/f^ mice after 4 weeks obese mEV treatment (*n* = 6 per group). All experiments were repeated at least twice with similar results. Data are presented as the mean ± SEM. *P* values are determined by unpaired two-side Student’s *t* test (**a**–**c**) or one-way ANOVA analysis (**d**–**h**). Source data are provided as a Source data file.

cGAS/STING signaling plays a critical role in sensing bacterial DNAs and initiating cellular inflammatory responses^32,33^. Thus, we further assessed whether obese mEVs induce β cell inflammatory responses through the cGAS/STING-mediated mechanism. Concomitant with accumulation of bacterial DNAs, there were greater levels of cGAS and phosphorylated STING within islets of 5wks HFD Vsig4^−/−^ mice than 5wks HFD WT mice (Fig. 7a). In addition, obese β cells had greater levels of cGAS and phosphorylated STING than that in lean β cells (Fig. S7a). Obese mEV treatment led to increased activation of cGAS/SITNG pathway in the islets of NCD Vsig4^−/−^ or C3^−/−^ mice (Figs. 7b and S7b,c). We also observed that islet macrophages isolated from lean WT mice showed elevated abundance of cGAS and IL1β after in vitro treatment with obese mEVs, whereas, obese mEVs treatment had minimal effects on islet macrophages isolated from lean Vsig4^−/−^ mice (Fig. S7d). However, GF EV or DNA-free EV treatment did not significantly affect the activation of cGAS/STING in the islets of NCD Vsig4^−/−^ recipient mice (Fig. S7e). The results from the Min6 cells treated with gut EVs also show that the accumulation of microbial DNAs led to the activation of cGAS/STING pathway (Fig. 7c). By contrast, the Min6 cells with depletion of cGAS or islets isolated from 8wks HFD cGAS^−/−^ mice had non-significant responses to obese mEV treatment (Figs. 7d, e and S7f–h). Similarly, obese mEVs treatment did not affect the siRNA-cGAS transfected islets isolated from patients with obesity (Figs. 7f and S7d, i). In addition, after 4 weeks of treatment, knockout of cGAS abolished the in vivo effects of obese mEVs on insulin secretion (Fig. 7g). Consistently, gut mEV treatment did not significantly affect β cell responses in β cell-specific STING knockout (InsCre^+^STING^f/f^) mice (Figs. 7h and S7j, k). Therefore, these results suggest that cGAS/STING signaling plays a critical role in microbial DNA-mediated β cell dysfunction.

## Discussion

In this study, we have shown that loss of Vsig4+ macrophages results in the translocation of intestinal EVs and microbial DNA accumulation within β cells, triggering obesity-associated islet inflammation and β cell abnormalities. By contrast, in healthy lean host, Vsig4+ macrophages residing in islets can prevent the infiltration of intestinal mEVs into β cells. Prolonged HFD feeding leads to a remarkable reduction in the population of Vsig4+ macrophages. Treatment of obese mEVs can initiate islet inflammation and decrease GSIS of NCD Vsig4^−/−^ mice, whereas NCD WT mice did not response to injection of obese mEVs. In addition, in vitro experiments show the importance of Vsig4+ macrophages in islets on preventing the effects of obese mEVs, as evidenced by minimal effect of mEVs on NCD WT islet responses but impaired insulin secretion of NCD Vsig4^−/−^ islets treated with obese mEVs. Depletion of complement component C3 blunted the ability of Vsig4+ macrophages to remove intestinal mEVs from circulation, thus demonstrating the critical role of C3 in the interaction between Vsig4+ macrophages and intestinal mEVs. Finally, we demonstrate that, among intestinal EVs, microbiota-derived bacterial DNA containing EVs are pathogenic to the development of islet inflammation and β cell dysfunction through activation of cGAS/STING signaling. Taken together, these studies indicate that microbial DNAs, as a result of intestinal mEV infiltration, promote obesity-associated islet inflammation and β cell abnormalities.

In the context of obesity, various microbiota-derived products can pass through disrupted gut barrier^7^. Previous studies have reported that bacterial DNA content was elevated in the circulation and metabolic tissues of both obese mice and patients with obesity^15–18^. In line with these findings, there were more bacterial DNAs accumulated in the β cells of both humans and mice with obesity. However, the source of circulating bacterial DNAs remains debated^7^. In current study, we found that intestinal EVs can be readily transported beyond gut lumen and microbial DNAs can be delivered into β cells in the context of obesity. By contrast, in lean WT recipient mice, PKH26 EVs did not penetrate through the intact gut barrier. While various mechanisms have been suggested for the impairment of gut barrier in obesity^7^, the mechanisms underlying the translocation of mEVs from the gut lumen into host circulation and distal tissues remain unknown.

Vsig4+ macrophages are the key effector cells blocking bacterial products from the circulation^17,30,31,34^. Consistently, we demonstrate that islet Vsig4+ macrophages protect β cell from the infiltration of intestinal mEVs, as demonstrated by very few amounts of microbial DNAs taken up by β cells after NCD WT islets treated with intestinal mEVs. In addition, we demonstrated that, in the absence of liver Vsig4+ macrophages, islet Vsig4+ macrophages exert profound protection from the pathogenesis of gut mEVs in the KC-depleted mice. We also observed that gut mEV treatment had minimal effects on systemic inflammation and metabolic phenotype in KC-KO mice, suggesting the importance of tissue-resident Vsig4+ macrophages on the maintenance of local inflammation. By contrast, depletion of Vsig4 or islet macrophages resulted in robust accumulation of microbial DNAs in islets after treatment of intestinal mEVs. There was remarkably decreased abundance of Vsig4+ macrophages in both obese mice and patients with obesity. This observation supports the notion that obesity increases the susceptibility of various organs to the bacterial infection, and now our data show that pancreatic β cells are susceptible targets in obesity. In addition to Vsig4, scavenger receptors play important role in bacterial phagocytosis in macrophages. Ono et al.^35^ reported that Kupffer cells can capture both Gram-positive and Gram-negative bacteria through a scavenger receptor A-dependent manner.

Earlier studies have proposed that complement component C3 is required for the ability of Vsig4+ macrophages to interact with circulating bacteria^17,30^. In agreement with the importance of complement component C3-mediated opsonization for the function of Vsig4+ macrophages, Vsig4+ macrophages failed to block the dissemination of injected intestinal mEVs in NCD C3^−/−^ mice, subsequently resulting in accumulation of microbial DNAs in islets and β cell abnormalities. Previous studies also suggest that C3 proteins play important roles in protecting β cells from the proinflammatory cytokines-induced stress^36^. In addition to this C3-dependent mechanism, Zeng et al. suggest that liver Vsig4+ macrophages can capture Gram-positive bacteria through directly binding with the LTA component of these bacteria^31^. Another study by Broadley et al. suggests that the scavenger receptors of Kupffer cells can rapidly clear *Listeria* from circulation before opsonization^34^. While we did not fully assess these mechanisms in current study, in the LTA-treated lean WT mice Vsig4+ macrophages still can efficiently block gut mEVs and there was no detectable bacterial DNA within the pancreas. In addition, our observation from NCD C3^−/−^ mice injected with PKH26-labled mEVs supports the critical role of C3-mediated opsonization in the interaction between Vsig4+ macrophages and intestinal EVs.

Emerging evidence shows that various microbial products exacerbate the tissue inflammation and metabolic responses of host in the context of obesity^7,17^. Previous studies have suggested that islet inflammation contributes to β cell abnormalities^3–5,37^. In current study, we found that infiltration of intestinal mEVs resulted in increased number of CD11c + islet macrophages and elevated levels of proinflammatory cytokines such as IL1β. While previous study has indicated accumulation of CD11c + islet macrophages promotes islet inflammation and subsequently impairs insulin secretion^3^. Based on our observation from the obese mEVs-treated lean Vsig4^−/−^ or C3^−/−^ mice, gut mEVs infiltration could be one of factors expanding the population of CD11c + islet macrophages in obesity. In addition, the results from the in vitro experiment with Min6 cells co-cultured with obese mEVs also demonstrate that intestinal mEVs can directly enhance the production of proinflammatory cytokine IL1β in β cells, concomitant with decreased insulin secretion. Previous studies suggest that the acute postprandial rise in IL1β production is necessary for meal-induced insulin secretion in a fasting-refeeding mouse model and is required for normal glycemic control^38^. However, chronic accumulation or elevated production of IL1β is pathogenic to β cell functions.

Microbiota can secrete various types of bacterial DNAs-containing EVs which are main components of intestinal EVs^17,25,39,40^. Previous studies have shown the profound regulation of microbiota-derived EVs in various diseases models^25,39,40^. We observed minimal effect of GF EVs on NCD Vsig4^−/−^ mice, thus demonstrating that microbiota-derived EVs contribute to the pathogenic effects of intestinal EVs. However, Jing et al.^41^ have reported that intestinal epithelial cell-derived EVs play a critical role in intestinal tract immune balance which can prevent the pathogenesis of inflammatory bowel disease.

Previous studies have demonstrated that microbial DNAs may contribute to tissue inflammation and metabolic disorders of patients with morbid obesity^16,17^. While EVs can harbor various cargos, we found that microbial DNAs are one of the key pathogenic molecules inducing islet inflammation and β cell abnormalities, as demonstrated by minimal effects of intestinal EVs on islet responses after depletion of microbiota-derived EVs or microbial DNAs. These observations were similar with a previous study that has demonstrated the pathogenesis of microbial DNAs on inflammation in insulin-target cells^17^. While gut mEVs are barely leaked out of intestinal lumen in lean/healthy humans and mice, previous studies have shown that gut mEVs from lean WT mice can cause cellular inflammation and insulin resistance^17^. Early studies have shown that outer membrane vesicles shed from gut microbiota contain polysaccharide or a variety of digesting enzymes that can significantly affect intestinal immune cell responses^42,43^. The discrepancy between our observation and these previous findings may be due to the EV types. Because there are marked differences in types and abundance of cargos carried by distinct types of EVs.

cGAS/STING signaling plays a critical role in sensing foreign DNAs and subsequently initiating cellular inflammatory responses^32^. Previous studies have shown the critical role of cGAS/STING pathway in detecting intracellular bacterial DNAs and activating inflammatory action^17,44,45^. In addition, Bai et al.^46^ have shown that the activation of cGAS/STING pathway in response to mitochondrial DNA release contributes to obesity-associated adipose tissue inflammation. Depletion of STING can also blunt liver inflammation and fibrotic activation^47^. We found that microbial DNAs enhanced levels of cGAS and phosphorylated STING, whereas knockout of cGAS can abolish the effects of intestinal mEVs on islet functions. Thus, cGAS/STING pathway is critical for microbial DNAs to trigger β cell inflammation. Emerging evidence indicates that the nucleotide-binding oligomerization domain (NOD) proteins exert profound function sensing bacterial peptidoglycans, g-d-glutamyl-meso-diaminopimelic acid, and/or muramyl dipeptide, resulting in the activation of proinflammatory responses of host cells^48^. However, whether microbial EVs contain sufficient amount of NOD ligands to activate NOD-associated signaling is still unknown.

In summary, we find that intestinal EVs encapsulating microbial EVs can pass through disrupted gut barriers and exacerbate obesity-associated islet inflammation and β cell abnormalities. Vsig4+ macrophages in islets play a critical role in clearing intestinal mEVs from circulation, whereas a reduction in the population of these cells is observed in obesity. Based on these studies, we suggest that recovery of Vsig4+ macrophages could attenuate microbial DNA-mediated islet inflammation and β cell abnormalities.

## Methods

### Animal care and use

All animal procedures complied with all relevant ethical regulations and were conducted under approved protocols (Protocol# S19147) by University of California, San Diego Research Guidelines for the Care and Use of Laboratory Animals, and all animals were randomly assigned to cohorts when used. cGAS^−/−^, C3^−/−^, ROSA26iDTR, Clec4f-Cre, MIPGFP, InsCre, and STING^f/f^ mice were received from the Jackson Laboratory. Vsig4^−/−^ mice (C57BL/6 J background) were maintained by Dr. Wei Ying lab in UCSD. Vsig4 wild-type (WT) mice were produced by crossing Vsig4 heterozygous mice together. All mice were maintained at 22–24 °C with a 12/12 h light-dark cycle and ad libitum access to diet and water. At 8 weeks of age, male mice were used as recipients for extracellular vesicle (EV) injection and fed ad libitum on a high-fat diet (HFD; 60% fat calories, 20% protein calories, and 20% carbohydrate calories; Cat. No. D12492, Research Diets) or a normal chow diet (NCD, Cat. No. 0001319, LabDiet) for various durations. Germ-free (GF) C57BL/6 mice were maintained in the UCSD Gnotobiotic Mouse Facility. GF mice were fed an autoclaved chow diet (Cat. No. 2019S, Teklad Global 19% Protein Extruded Rodent Diet; Harlan Laboratories) or an irradiated sterilized 60% HFD (Cat. No. 06414, Teklad). To test the effect of saturated fatty acid on the population of Vsig4+ macrophages in pancreas, a group of NCD WT mice was administrated with palmitate acid (500 μM PA mixed with 200 μL corn oil) through daily oral gavage for 2 weeks. To deplete Kupffer cells (KC), diphtheria toxin (DT) was intraperitoneally (i.p.) injected into Clec4fCre+DTR + lean mice (KC-KO) for three days (200 ng/mouse), and these mice were treated with DT (200 ng/mouse) every 2 days to prevent KC recovery. To test the effects of liposaccharide (LPS) and gut mEVs on metabolic phenotypes, lean Vsig4^−/−^ mice were i.p. injected with LPS (300 ng/g of body weight) every 2 days.

#### Isolation of islets

NCD and HFD mice were euthanized, and freshly-prepared collagenase P solution (0.5 mg/ml; passed through a 0.2 μm pore-size filter) was injected into the pancreas via the common bile duct. The perfused pancreas was digested at 37 °C for 10 min, and the islets were handpicked under a stereoscopic microscope. To sort out GFP + β cells, islets were dispersed into single-cell suspensions, and then cells with fluorescent reporters were purified using a SONY MA900 flow cytometer (SONY).

#### In vivo and in vitro EV treatment

For in vitro GSIS assays, 1 × 10^7^ EVs on the basis of NanoSight analysis were added to 10 islets for 24 h. For in vivo treatment, recipient mice were tail vein injected with 5 × 10^9^ EVs twice per week. All experiments were repeated at least twice independently.

### In vivo glucose-stimulated insulin secretion assay

Mice received one dose of dextrose (1 g/kg body weight) via i.p. injection after 12 h of fasting. Plasma were collected from the retro-orbital vein after 10 mins of glucose injection. Insulin levels were measured in these plasma samples.

### EV purification and characterization

The intestinal EVs were prepared from small intestine lumen contents of either NCD or HFD mice. Debris and dead cells in the lumen contents were removed by centrifugation at 1000 × *g* for 10 min and then filtrated through a 0.2 µm filter. The supernatant was then subjected to ultracentrifugation at 100,000 × *g* for 4 h at 4 °C with a Type 70 Ti fixed-angle rotor (Beckman Coulter). The EV-containing pellet was resuspended in 1 ml sterile PBS and passed through a 0.2 µm filter to remove large particles. The particle size and concentration of intestinal EVs were measured by NanoSight analysis (Malvern Instruments). For electron microscopy, EVs were fixed with 2% paraformaldehyde and loaded on Formvar and carbon-coated copper grids. Then the grids were placed on 2% gelatin at 37 °C for 20 min, rinsed with 0.15 M glycine/PBS and the sections blocked using 1% cold water fish-skin gelatin. Grids were viewed using a JEOL 1200EX II (JEOL) transmission electron microscope and photographed using a Gatan digital camera (Gatan). To monitor EV trafficking, EVs were labeled with PKH26 fluorescent dye using the PKH26 fluorescent cell linker kit (Cat. No. PKH26GL-1KT, Sigma). After PKH26 staining, the EVs were washed with PBS and collected by ultracentrifugation (100,000; × *g* for 2 h) at 4 °C. Finally, PKH26-labeled EVs were resuspended in sterile PBS.

In all, 1 mL of plasma were pooled from either NCD WT or 16wks HFD WT mice via cardiac puncture in a sterile hood. To collect EVs from plasma, plasma (0.5 mL) was diluted with 2 mL sterile PBS and then passed through a 0.2 µm filter. The supernatant was then subjected to ultracentrifugation at 100,000 × *g* for 4 h at 4 °C with a SW60 Ti swinging-bucket rotor (Beckman Coulter). After ultracentrifugation, both plasma EV pellets and EV-free supernatant were used to measure the abundance of bacterial DNAs. The rest of the plasma (0.5 mL) were diluted with 0.5 mL of sterile PBS and then passed through a 0.2-µm filter and finally used to measured bacterial DNA content. To evaluate the levels of external bacterial DNA contamination, we also processed plasma samples from obese germ-free mice and then measure 16s rRNA abundance by qPCR analysis.

### In vivo EV trafficking assays

PKH26-labeled EVs (5 × 10^9^ EVs per mouse) were delivered to either NCD or HFD recipient mice through either injection into tail vein or jejunum section. After 16 h EV injection, parts of pancreas were collected for detecting the appearance of PKH26 red fluorescence.

### Effect of lipoteichoic acid on the ability of Vsig4 to bind gut mEVs

Lean WT and KC-KO mice were intravenously injected with purified LTA (from *S. aureaus*, Sigma; 1 mg per mouse). After 16 h, PKH26-labeled obese mEVs (5 × 10^9^ EVs/mouse) were intravenously injected into these LTA-treated mice. Pancreatic sections were prepared from these mice after 16 h injection of PKH26 EVs.

### Depletion DNA of intestinal EVs

The intestinal EV pellet was dissolved in 100 µL PBS. As previously described, these EVs were loaded into a Gene Pulser/micropulser Cuvettes (Bio-Rad) for electroporation (GenePulser Xcell electroporator, Bio-Rad) and then treated with DNase I (300U) for 30 mins, 37 °C.

### Depletion of islet macrophages

Islets were treated with CSF1R (CD115) antibody (BioXCell; 10 μg/10-30 islets) to deplete macrophages.

### Quantification of bacterial DNA using real-time PCR

Levels of bacterial DNAs were assessed by qPCR using a Femto Bacterial DNA Quantification Kit by following the manufacturer’s instructions. Briefly, Bacterial DNAs were extracted from EVs or plasma using the ZymoBIOMICS DNA extraction kits according to the manufacturer’s instructions. The concentration of bacterial DNA in each sample was determined from the standard curve using a nonlinear regression four-parameter variable slope analysis.

### Human biospecimens

The UCSD IRB has waived the review and approval for the usage of human islets (Prodo Laboratories; purity > 90%; Islet purity was determined by staining with Diphenylthiocarbozone to distinguish islets from non-islet tissue.) or pancreatic samples (Novus Biologicals or International institute for the advancement of medicine) (UCSD IRB exempt protocol# 801547) due to the secondary usage of existing human pancreatic biospecimens. The information related to human islets used in this study is provided in Supplementary table 1.

Archival human plasma samples used in this study were from generally healthy, overnight-fasted lean men and individuals with obesity residing in San Diego County. Samples were collected in accordance with the Declaration of Helsinki and the principles of Good Clinical Practice as part of the Community of Mine Study, approved by the UC San Diego Institutional Review Board^49^. All participants provided written informed consent. Body mass index values are presented in Supplementary Table 2.

#### Glucose-stimulated insulin secretion assays

Primary mouse islets isolated from both lean and obese mice, human islets, or Min6 cells were used to evaluate the effects of intestinal EVs or siRNA-cGAS on GSIS. To measure static GSIS, after washing twice with 1 g/L glucose DMEM, islets were incubated overnight in 1 g/L glucose DMEM, at 37 °C, 5% CO_2_. Next day, the islets were washed with fresh 2.8 mM glucose Krebs Ringer Bicarbonate buffer (KRB buffer; 2.6 mM CaCl_2_/2H_2_O, 1.2 mM MgSO_4_/7H_2_O, 1.2 mM KH_2_PO_4_, 4.9 mM KCl, 98.5 mM NaCl, and 25.9 mM NaHCO_3_, supplemented with 20 mM HEPES and 0.2% BSA), and then fasted with 2.8 mM glucose KRB buffer for 30 min. Next, islets were incubated for 60 min in 2.8 mM or 16.3 mM glucose KRB buffer. Insulin concentrations in the supernatant were determined using Ultrasensitive mouse insulin ELISA kits (Alpco, Cat. No. 80-INSHU-E01.1). To calculate the relative insulin secretion for each group, the glucose-stimulated insulin secretion data were normalized to total insulin content of the islets or Min6 cells. In another set of islets or Min6 cells, after treatment with siRNA-cGAS or intestinal EVs, total insulin content was measured and normalized to total protein of these cells.

### siRNA transfection

siRNA-cGAS (Cat. No. J-015607-17-0002 or J-055608-09-0002, Horizon discovery; 20 pM siRNA/10 islets or 0.1 × 10^6^ Min6 cells) was transfected into recipient cells with the lipofectamine RNAiMAX reagent. siRNA-cGAS was mixed with RNAiMAX reagent and then incubated at room temperature for 15 mins. This mixture was added into the medium of islets or Min6 cells. After 24 h, GSIS or inflammation in these cells were measured.

### Immuno-fluorescence staining

Parts of pancreas of NCD or HFD mice were snap frozen in optimum cutting temperature (O.C.T., Fisher Healthcare) with dry ice. Six µm cryo-sections of tissue sections were cut and fixed with pre-cold acetone for 20 min. Immunostaining was performed as previously described. Slides were blocked with 5% normal donkey serum for 60 min at RT. Consequently, the samples were incubated with anti-Vsig4 or anti-insulin antibody diluted 1:100 in PBS at 4 °C overnight. After washing, nuclei were stained with DAPI (4′,6-Diamidino-2-28 phenylindole dihydrochloride) for 10 min at room temperature. Mounting media and cover slips were then added to slides for imaging. Images were acquired on a Keyence Fluorescent Microscope, and were processed with ImageJ (NIH, Bethesda, MD).

### RNAscope in situ hybridization combined with immunofluorescence

We performed RNAscope in situ hybridization (ISH) to detect 16 s rRNA. Human pancreas sections (5 µm) were obtained from the international institute for the advancement of medicine and Novus Biologicals. Mouse pancreas were snap frozen in O.C.T with dry ice. Ten µm cryo-sections of tissue sections were cut and fixed with 4% PFA for 15 min at 4 °C, and finally dehydrated with 50%, 70%, and 100% ethyl alcohol gradients for 5 min each at room temperature. Tissue sections were then treated by hydrogen peroxide and protease IV at room temperature for 10 min each. 16 s RNA probes (Cat. No. 464461, Advanced Cell Diagnostics) were then added for 2 h at 40 °C. Signal amplification and detection reagents were applied sequentially and incubated in AMP 1, AMP 2, AMP 3, HRP-C1 (RNAscope® Multiplex fluorescent reagent kit v2, Cat. No. 323100, Advanced Cell Diagnostics), Opal 520 (Cat. No. PNFP1487001KT, Akoya Biosciences), or Opal 690 (Cat. No. FP1497001KT, Akoya Biosciences). Then, samples were immediately processed for immunofluorescence, and images were captured using Leica SP8 Confocal microscope.

### Flow cytometry analysis

Islets were dispersed and then stained with fluorescence-tagged antibodies to detect GFP + cells or CD11c + F4/80+ macrophages. These cells were analyzed or sorted out by MA900 flow cytometer (SONY). All antibodies were used with 1:200 dilution. Data were analyzed using Flowjo software.

### Quantitative reverse transcriptase-polymerase chain reaction analysis

Total RNA was extracted using the RNA extraction protocol according to the manufacturer’s instructions. cDNA was synthesized using SuperScript III and random hexamers (High-capacity cDNA reverse transcription kit, Cat. No. 4368813, ThermoFisher Scientific). qPCR was carried out in 10 μl reactions using iTaq SYBR Green supermix (Cat. No. 172-5125, Bio-Rad) on a StepOnePlus Real-Time PCR Systems (ThermoFisher Scientific). The data presented correspond to the mean of 2^−ΔΔCt^ from at least three independent experiments after being normalized to β-actin.

### Western blot analysis

Cells or tissues were homogenized in RIPA buffer supplemented with protease and phosphatase inhibitors. Equal amounts of cell lysate proteins (30 μg protein per lane for pSTING, CD63, or HSP70 detection, 10 μg protein per lane for cGAS or Vsig4 detection) from each biological replicate were subjected to western blotting. Using ChemiDoc XRS imaging system (BioRad), the protein bands on blots were detected with the SuperSignal West Femto Maximum Sensitivity Chemiluminescent Substrate (Cat. No. 34095, ThermoFisher Scientific). After detecting pSTING or Vsig4 (Figs. 2f and 2g), blots were incubated with the Restore^TM^ Western blot Stripping buffer (Cat. No. 21059, ThermoFisher Scientific) and then used for detecting STING or GADPH. Protein bands were analyzed using Image Lab software (BioRad). We normalized phosphorylated protein to total protein bands, or normalized protein expression to housekeeping protein bands. Western blot data in figures and supplemental figures are all representative of more than two independent experiments. GAPDH (Cat. No. 2118 S; 1:2000 dilution), pSTING (Cat. No. 72971; 1:2000 dilution), STING (Cat. No. 50494; 1:2000 dilution), and cGAS (Cat. No. 316595; 1:2000 dilution) antibodies were obtained from Cell Signaling Technology. Vsig4 (Cat. No. 17-5752-82; 1:2000 dilution) and HSP70 (Cat. No. MA5-31961; 1:2000 dilution) antibodies were received from ThermoFisher. HSP90 antibody (Cat. No. sc-101494; 1:2000 dilution) was obtained from Santa Cruz Biotechnology.

#### Statistical analysis

Tests used for statistical analyses are described in the figure legends. To assess whether the means of two groups are statistically different from each other, unpaired two-tailed Student’s *t* test was used for statistical analyses using Prism8 software (GraphPad software v8.0; Prism, La Jolla, CA). One-way ANOVA with Tukey multiple comparisons was used to compare the means of multiple groups. *P* values of 0.05 or less were considered to be statistically significant.

### Reporting summary

Further information on research design is available in the Nature Research Reporting Summary linked to this article.

## Supplementary information

Supplementary Information

Reporting summary

## Data Availability

The datasets generated in current study are available from the corresponding author upon reasonable request. There are no restrictions on data availability. Mouse lines in this study are available from the Jackson Laboratory. Source data are provided with this paper.

## Source data

Source Data
