# Supplementary material for: Accumulation of microbial DNAs promotes to islet inflammation and β cell abnormalities in obesity in mice
Source: Nat Commun. 2022 Jan 28;13:565. doi: 10.1038/s41467-022-28239-2 (PMC8799656; doi:10.1038/s41467-022-28239-2)
Supplement: Supplementary file 1 — Supplementary Information [file 41467_2022_28239_MOESM1_ESM.pdf]

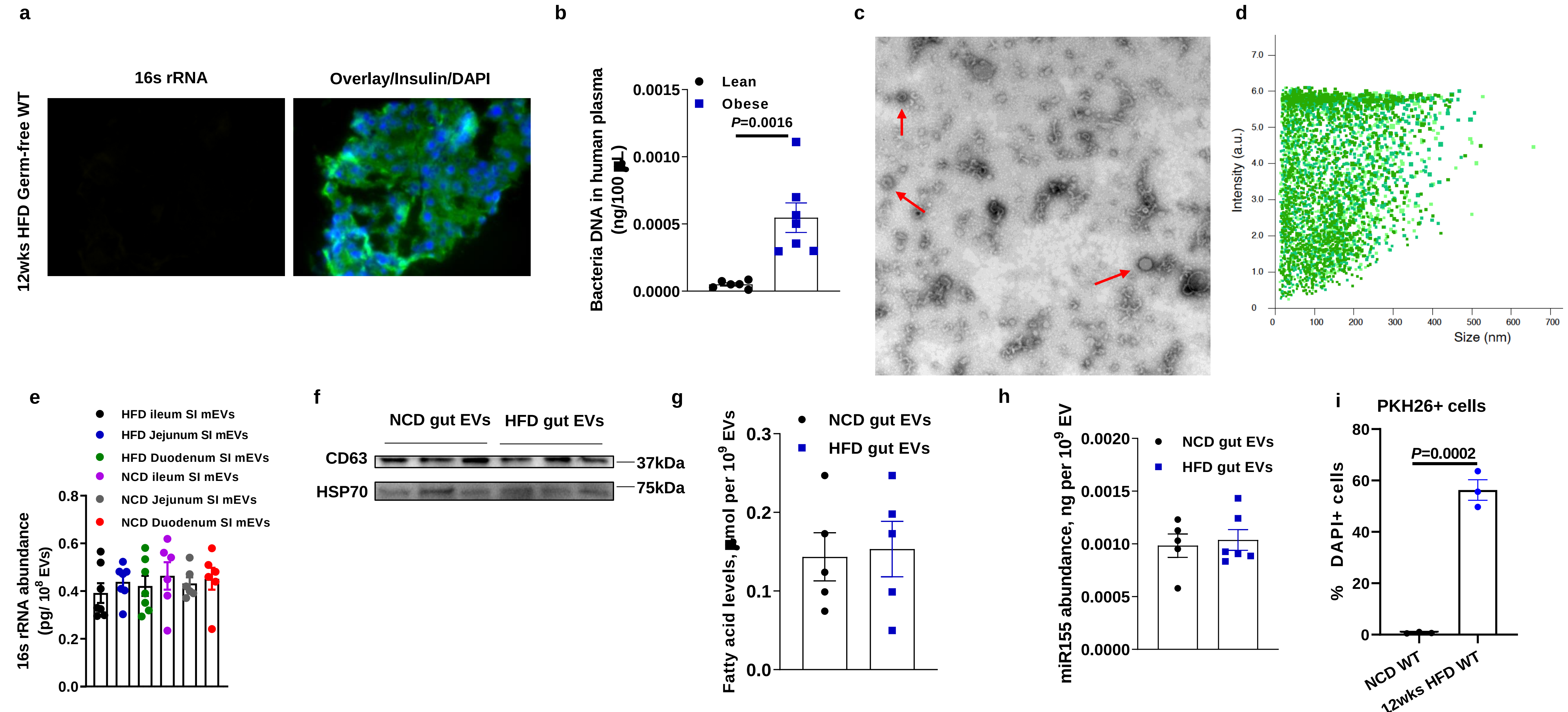

Supplementary Figure 1. Characteristics of intestinal extracellular vesicles (EVs). (**a**) 16s rRNA abundance in the pancreas of 12wks HFD germ-free WT mice. (**b**) 16s rRNA abundance in human plasma (n=6 of lean, n=7 of obese). Electron microscopy analysis (**c**; red arrows point EVs.) and NanoSight analysis (**d;** a.u.=arbitrary units) of intestinal EVs. (**e**) Bacterial DNA content within EVs collected from distinct small intestine sections (**e,** n=6 per group), EV marker expression (**f**), fatty acid levels (**g,** n=5 per group), and proinflammatory gene miR-155 abundance (**h,** n=5 of NCD gut EVs, n=6 of HFD gut EVs) within gut EVs of normal chow diet (NCD) or high fat diet (HFD)-fed mice. (**i**) Population of PKH26+ cells in the pancreas of lean or obese WT recipients after injection with PKH26-labled obese mEVs (n=3 per group). All experiments were repeated at least twice with similar results. Data are presented as the mean ± SEM. *P* values are determined by unpaired two-sided Student's *t* test (**i**). Source data are provided as a Source data file.

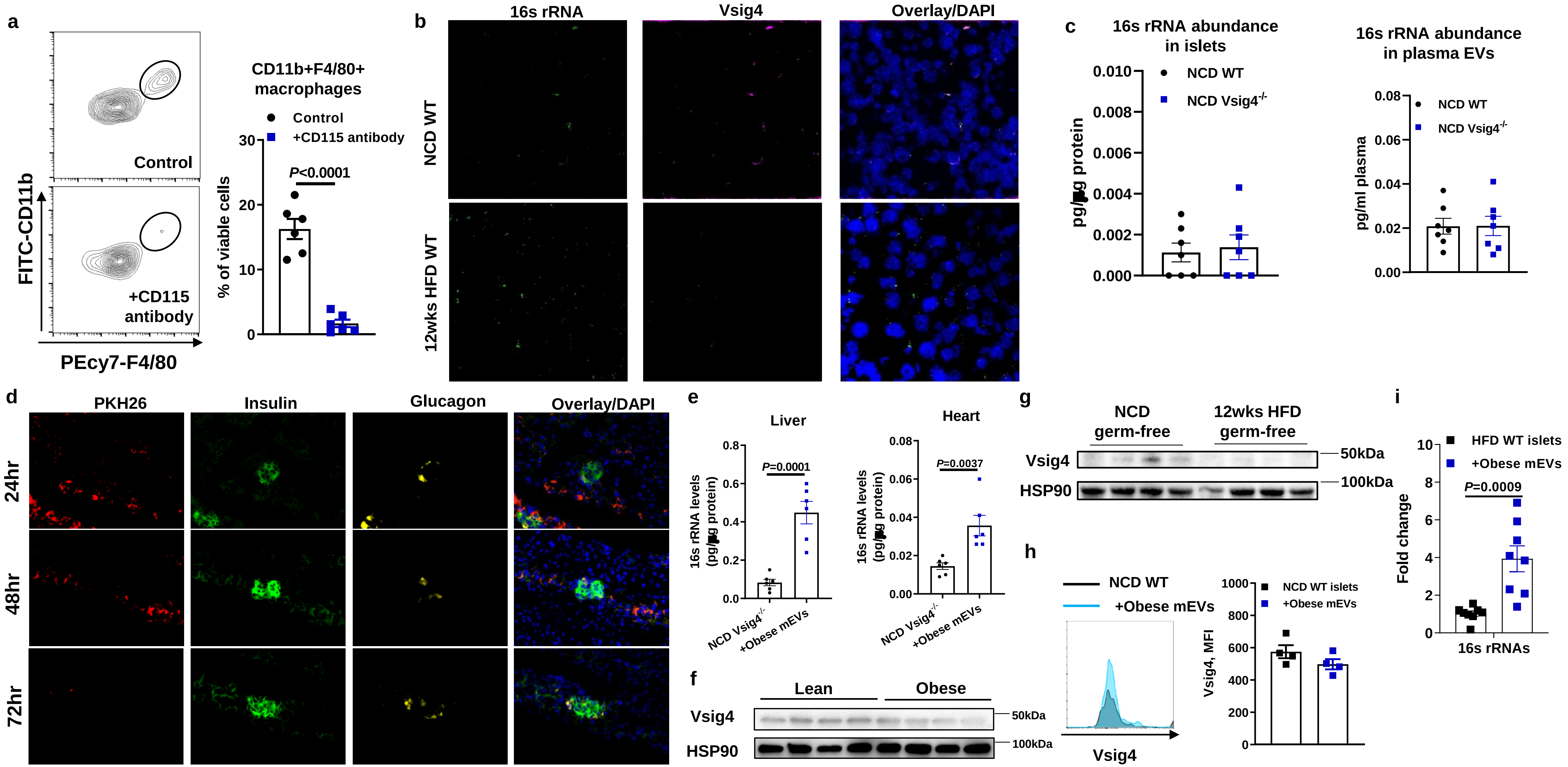

Supplementary Figure 2. Effect of HFD feeding on Vsig4 expression. (**a**) Macrophage population in lean WT islets after treatment with CD115 antibody (n=6 per group). (**b**) The expression of 16s rRNA and Vsig4 in the pancreas of both NCD WT and 12 weeks (wks) HFD fed WT mice. Representative images are from three independent experiments. (**c**) Bacterial DNA level in islets or plasma EVs of lean WT vs. Vsig4$^{-/-}$ mice (n=7 per group). (**d**) The appearance of PKH26 signals in the pancreas of NCD Vsig4$^{-/-}$ mice after intravenously injected with PKH26 labeled mEVs for 24, 48, or 72 hours. (**e**) qPCR analysis of 16s rRNA abundance in liver and heart of NCD Vsig4$^{-/-}$ mice after 4wks treatment with obese mEVs (n=6 per group). (**f**) Vsig4 abundance in human islets. (**g**) Vsig4 abundance in the pancreas of both lean and 12wks HFD fed germ-free mice. (**h**) Effect of obese mEV treatment on Vsig4 abundance in the islets of NCD WT recipient mice (n=4 per group). (**i**) Bacterial DNA levels within obese WT islets treated with obese mEVs (n=8 per group). All experiments were repeated at least twice with similar results. Data are presented as the mean ± SEM. *P* values are determined by unpaired two-sided Student's *t* test (**a, e, i**). Source data are provided as a Source data file.

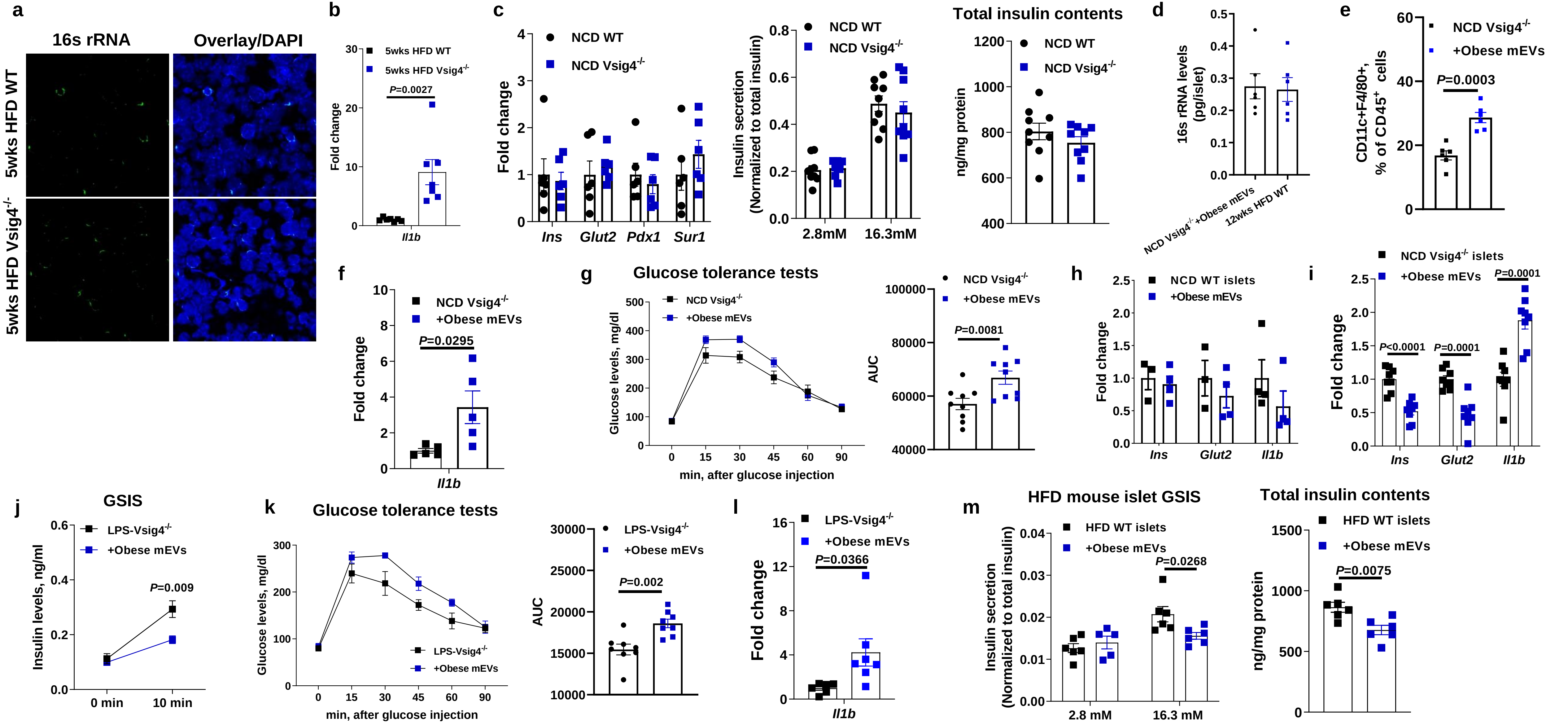

Supplementary Figure 3. Effects of Vsig4 depletion on islet inflammation and β cell abnormalities. (**a**) The abundance of 16s rRNAs within the pancreas of both 5wks HFD WT and 5wks HFD Vsig4$^{-/-}$ mice. Representative images are from three independent experiments. (**b**) *Il1b* expression in the islets isolated from 5wks HFD WT or 5wks HFD Vsig4$^{-/-}$ (n=6 per group). (**c**) Comparison of β cell functions between lean WT vs. Vsig4$^{-/-}$ mice (n=5-8 per group). (**d**) qPCR analysis of 16s rRNA abundance in islets of mEV-treated NCD Vsig4$^{-/-}$ and 12wks HFD WT mice (n=5 per group). (**e**) The population of CD11c+F4/80+ islet macrophages of NCD Vsig4$^{-/-}$ recipient mice after 4 weeks of obese mEV treatment (n=6 per group). *Il1b* abundance in the islets (**f,** n=5 per group) and glucose tolerance (**g,** n=9 per group) of NCD Vsig4$^{-/-}$ injected with obese mEVs. After 4 weeks of obese mEV treatment, the expression of *Il1b* and key genes associated with insulin synthesis and secretion in the islets isolated from NCD WT mice (**h,** n=4 per group) or NCD Vsig4$^{-/-}$ mice (**i,** n=8 per group). Effects of obese mEVs on *in vivo* GSIS (**j,** n=5 per group), glucose tolerance (**k,** n=8 per group), and islet *Il1b* abundance (**l,** n=7 per group) in the liposaccharide (LPS) treated lean Vsig4$^{-/-}$ mice. (**m**) Effects of obese mEV treatment on the glucose-stimulated insulin secretion (GSIS) and cellular insulin content of obese islets (n=5-6 per group). All experiments were repeated at least twice with similar results. Data are presented as the mean ± SEM. *P* values are determined by unpaired two-sided Student's *t* test (**b, e, f, g, i-m**).

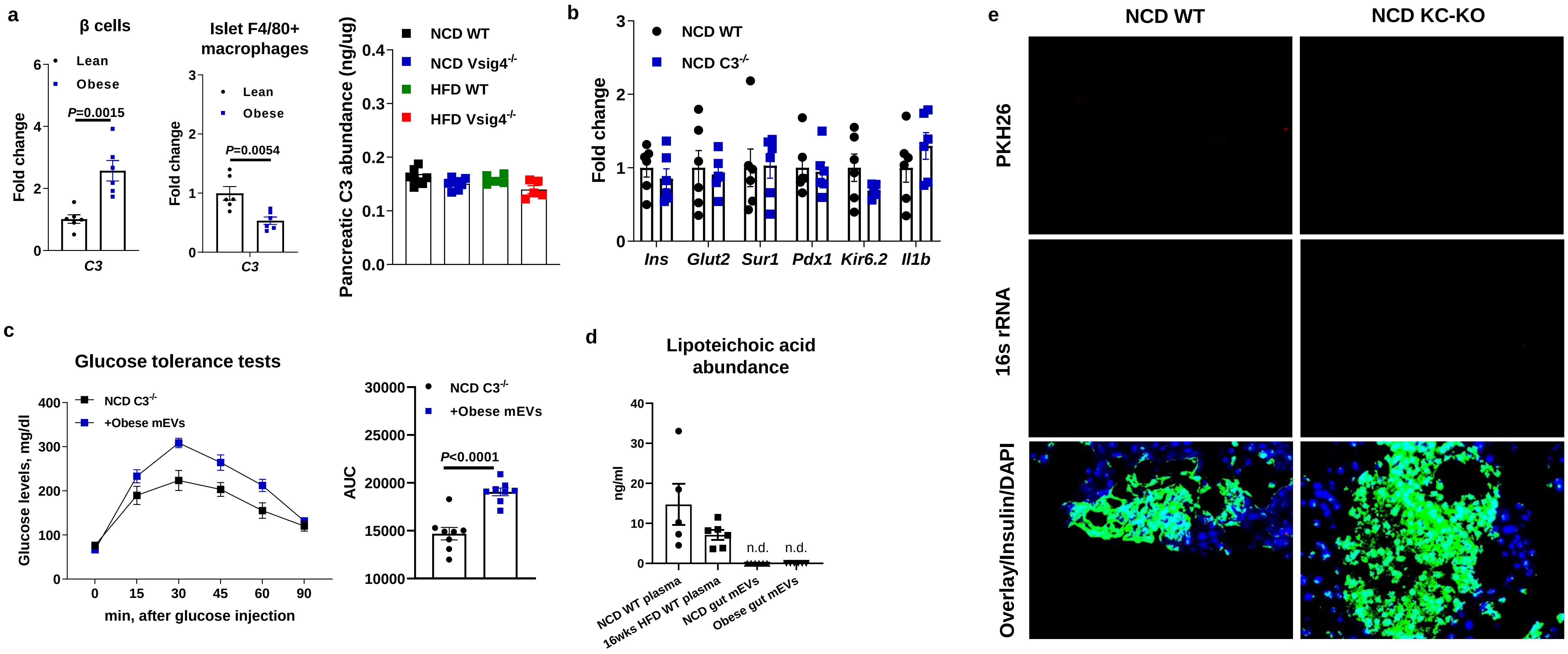

Supplementary Figure 4. Effects of complement component 3 (C3) knockout on interaction between Vsig4+ macrophages and mEVs. (**a**) C3 levels within the pancreas of NCD or 12wks HFD-fed mice (For C3 gene expression, n=6 per group; for pancreatic C3 abundance, n=8 of NCD WT or Vsig4$^{-/-}$, n=5 of HFD WT or Vsig4$^{-/-}$). (**b**) The abundance of *Il1b* and key genes related to insulin secretion and production of lean WT vs. C3$^{-/-}$ mice (n=6 per group). (**c**) The glucose tolerance of NCD C3$^{-/-}$ mice injected with obese mEVs (n=8 per group). (**d**) The levels of lipoteichoic acid (LTA) in the circulation and gut mEVs (n=5-6 per group). n.d., non-detectable. (**e**) The present of red fluorescent signal in the pancreas of lean WT and diphtheria toxin-treated NCD Clec4fCre+DTR+ mice (KC-KO) after both LTA and PKH26 gut mEV injection. Representative images are from three independent experiments. All experiments were repeated at least twice with similar results. Data are presented as the mean ± SEM. *P* values are determined by unpaired two-sided Student's *t* test (**a** and **c**).

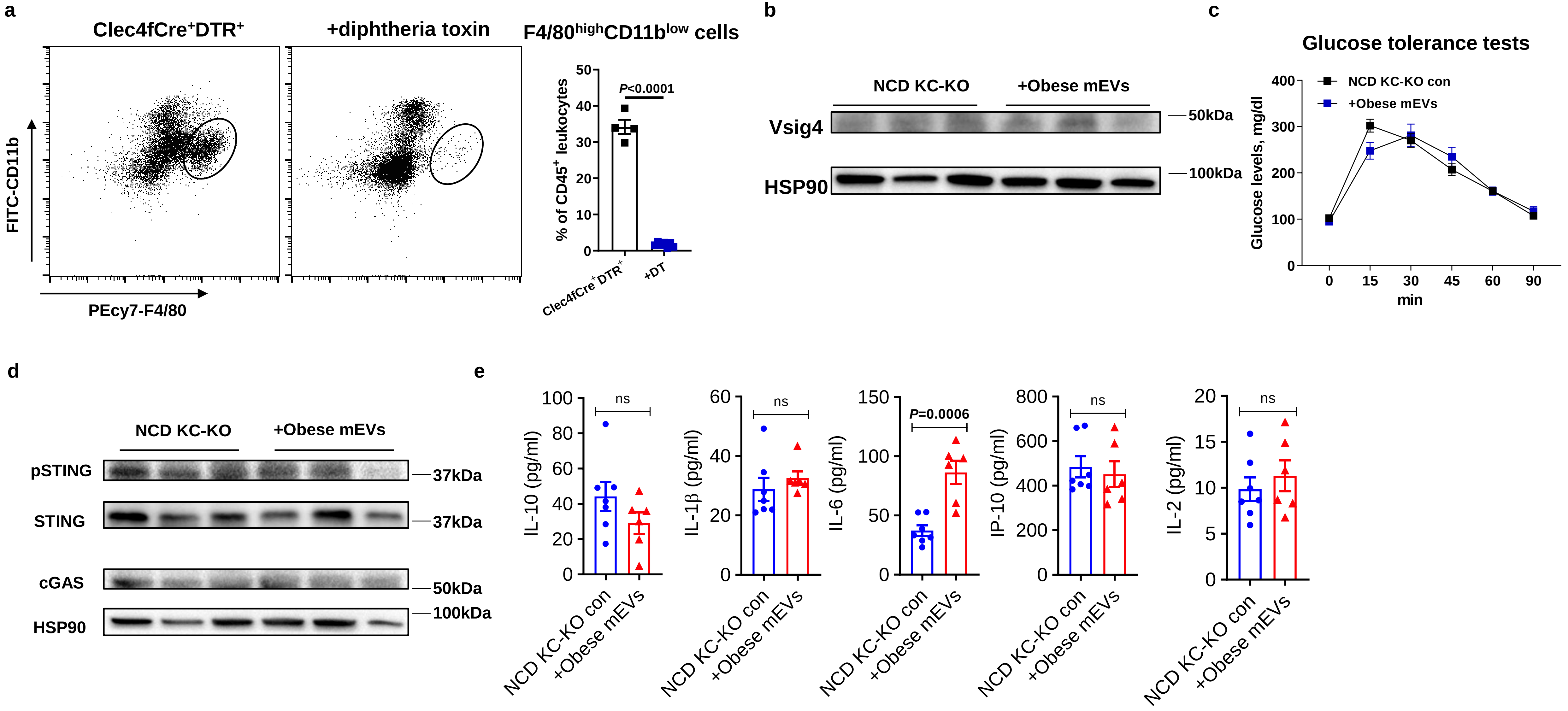

Supplementary Figure 5. Effects of Kupffer cell depletion on the infiltration of gut mEVs into islets. (**a**) FACS analysis of F4/80^{high}CD11b^{low} cells (Kupffer cells) in the liver of lean Clec4fCre+DTR+ mice after injected with diphtheria toxin (KC-KO) (n=4 per group). Effect of obese mEV treatment on islet Vsig4 abundance (**b**) and glucose tolerance (**c,** n=8 per group) of lean KC-KO mice. (**d**) The abundance of cGAS and phosphorylated STING in the islets of lean KC-KO after 4 weeks treatment with obese mEVs. (**e**) Effects of obese mEVs on the levels of inflammation-associated cytokines in the circulation of lean KC-KO mice (n=7 of KC-KO con, n=6 of KC-KO+Obese mEVs). ns, non-significant. All experiments were repeated at least twice with similar results. Data are presented as the mean ± SEM. *P* values are determined by unpaired two-sided Student's *t* test (**a, c, e**). Source data are provided as a Source data file.

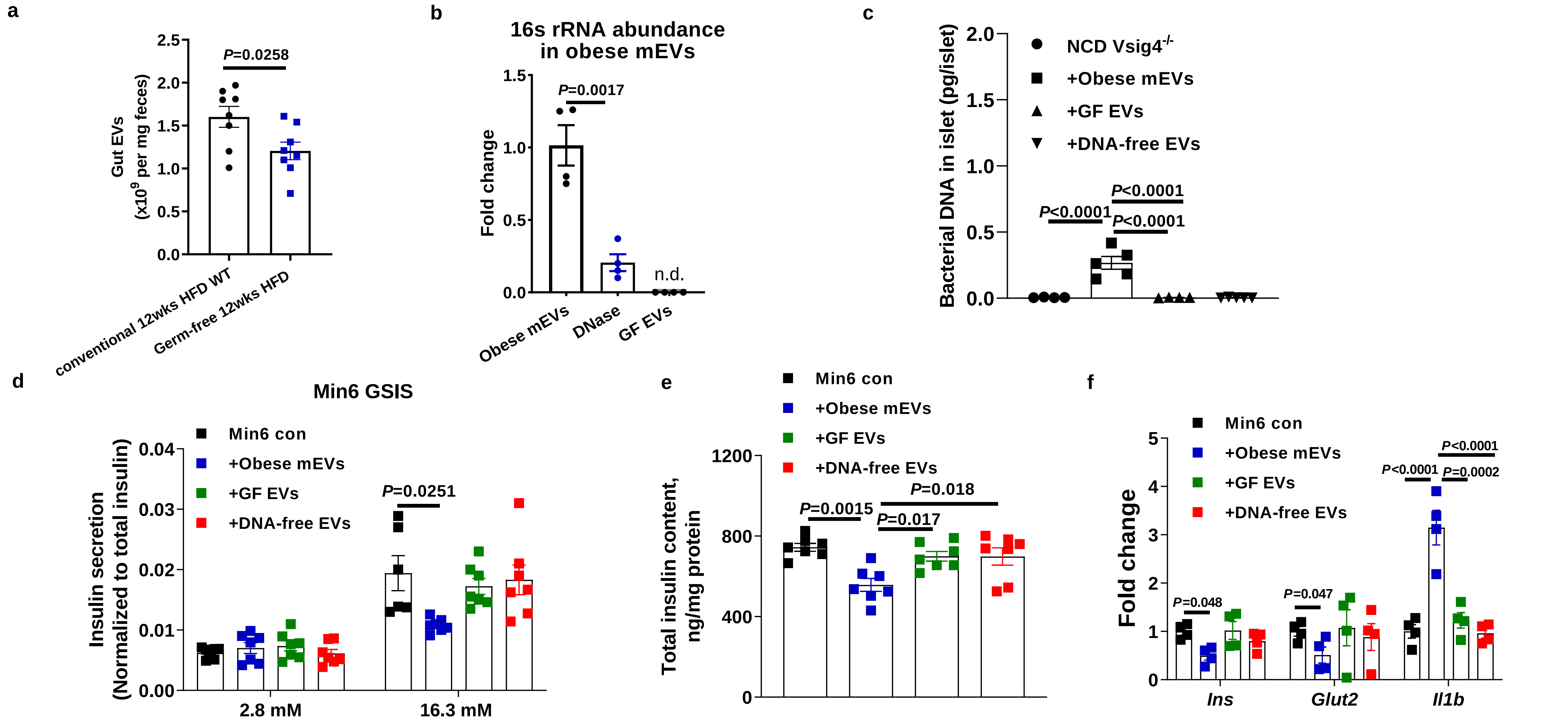

Supplementary Figure 6. Effects of DNA depletion on the ability of obese mEVs to regulate islet responses. (**a**) Comparison of EV numbers yield from the intestinal feces collected from 12wks HFD conventional or germ-free (GF) WT mice (n=8 per group). (**b**) The 16s rRNA abundance within intestinal EVs after electropolarization and DNase treatment or germ-free gut EVs (GF EVs) (n=4 per group). n.d., nondetectable. (**c**) qPCR analysis of 16s rRNA abundance within islets of lean Vsig4$^{-/-}$ mice treated with gut EVs (n=4-5 per group). Effects of intestinal EVs on GSIS (**d,** n=6-7 per group), total insulin content (**e,** n=7 per group), and the expression of insulin production and secretion-related genes (**f,** n=4 per group) of Min6 cells. All experiments were repeated at least twice with similar results. Data are presented as the mean ± SEM. *P* values are determined by unpaired two-sided Student's *t* test (**a** and **b**) or one-way ANOVA (**c-f**).

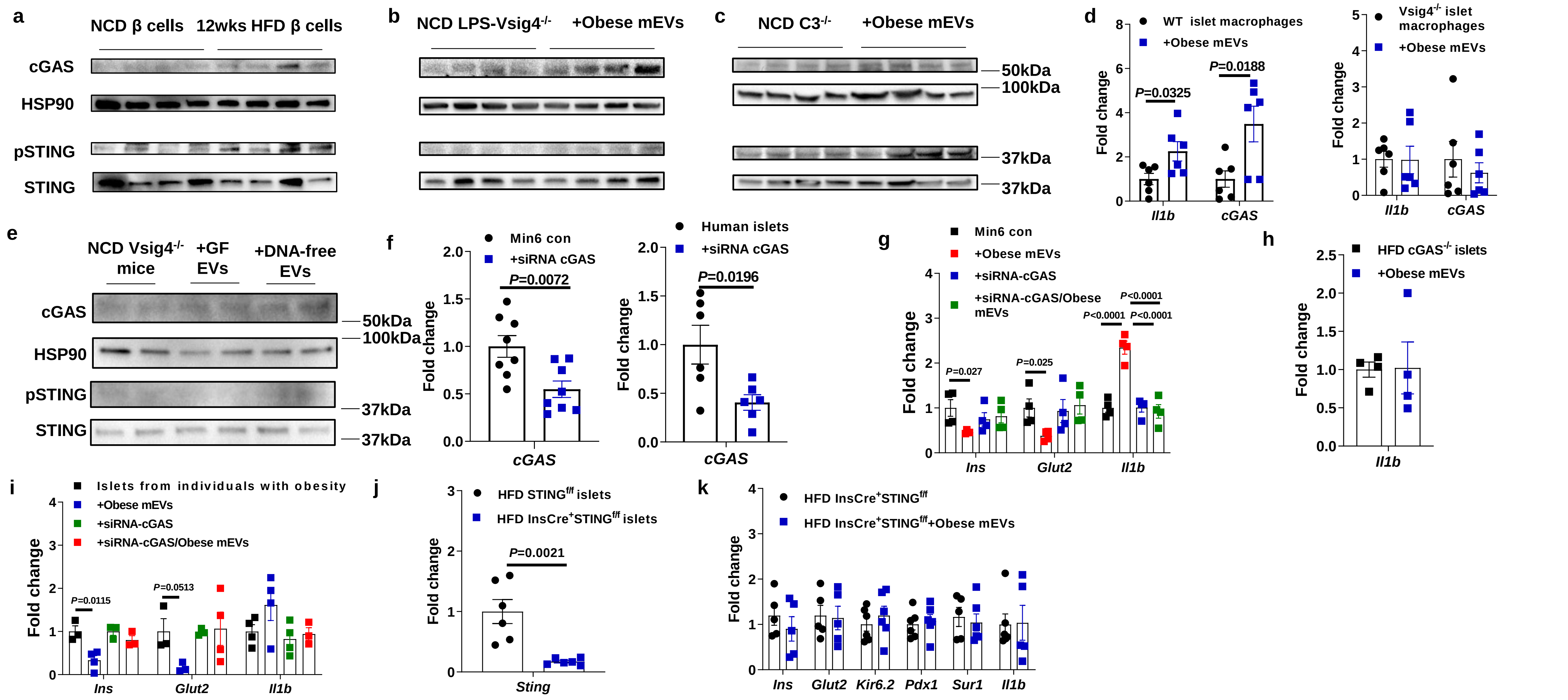

Supplementary Figure 7. The regulation of cGAS/STING signaling on microbial DNA-mediated β cell abnormalities. (**a**) The activation levels of cGAS/STING in β cells isolated from lean or 12wks HFD MIPGFP mice. The levels of cGAS, phosphorylated STING, and total STING protein in the islets of NCD LPS-treated Vsig4$^{-/-}$ (**b**) or C3$^{-/-}$ (**c**) mice after 4 weeks of obese mEV treatment. (**d**) The activation of either lean WT or Vsig4$^{-/-}$ islet macrophages after *in vitro* treatment with obese mEVs (n=6 pre group). (**e**) Effects of obese germ-free intestinal EVs (GF EVs) and DNA-free intestinal EVs on the activation of cGAS/STING signaling in the islets of NCD Vsig4$^{-/-}$ mice. (**f**) cGAS abundance after transfection of siRNA-cGAS (n=8 per group). Effect of obese mEVs on the abundance of *Ins*, *Glut2*, and *Il1b* in the siRNA cGAS treated Min6 cells (**g,** n=4 per group) or the islets (**h,** n=4 per group) isolated from 8wks HFD cGAS$^{-/-}$ mice. (**i**) The expression of *Il1b* and key genes associated with insulin synthesis and secretion in siRNA-cGAS and/or obese mEVs treated islets derived from humans with obesity (n=4 per group). (**j**) Sting abundance in the islets of 8wks HFD STING$^{f/f}$ and InsCre^{+}STING$^{f/f}$ mice (n=6 per group). (**k**) The abundance of key genes associated with β cell responses in 8wks HFD STING$^{f/f}$ or InsCre^{+}STING$^{f/f}$ mice injected with obese mEVs (n=5 per group). All experiments were repeated at least twice with similar results. Data are presented as the mean ± SEM. *P* values are determined by unpaired two-sided Student's *t* test (**d, f-k**). Source data are provided as a Source data file.

Supplementary table 1. Characteristics of human pancreas or islets used in this study.												
**Human islet/pancreas preparation**	**1**	**2**	**3**	**4**	**5**	**6**	**7**	**8**	**9**	**10**	**11**	**12**
BMI (kg/m^2)	27.3	31.9	32.8	26.6	28	33.6	27.2	34.6	N/A	N/A	31.25	33.75
age (years)	21	22	56	41	41	46	40	62	64	68	47	49

Supplementary table 2. Body mass index.	
	**BMI**
Individual with obesity & non-diabetes	30.39
Individual with obesity & non-diabetes	32.71
Individual with obesity & non-diabetes	35.28
Individual with obesity & non-diabetes	35.87
Individual with obesity & non-diabetes	35.05
Individual with obesity & non-diabetes	36.88
Individual with obesity & non-diabetes	31.96
Lean individual without diabetes	20.49
Lean individual without diabetes	22.79
Lean individual without diabetes	23.23
Lean individual without diabetes	23.59
Lean individual without diabetes	24.5
Lean individual without diabetes	23.26
