# Supplementary material for: Accumulation of microbial DNAs promotes to islet inflammation and β cell abnormalities in obesity in mice
Source: Nat Commun. 2022 Jan 28;13:565. doi: 10.1038/s41467-022-28239-2 (PMC8799656; doi:10.1038/s41467-022-28239-2)
Supplement: Supplementary file 2 — Reporting summary [file 41467_2022_28239_MOESM2_ESM.pdf]

nature research

Corresponding author(s): Wei Ying

Last updated by author(s): Dec 16, 2021

Reporting Summary

Nature Research wishes to improve the reproducibility of the work that we publish. This form provides structure for consistency and transparency in reporting. For further information on Nature Research policies, see our Editorial Policies and the Editorial Policy Checklist.

Statistics

For all statistical analyses, confirm that the following items are present in the figure legend, table legend, main text, or Methods section.

n/a	Confirmed	
☐	☒	The exact sample size (n) for each experimental group/condition, given as a discrete number and unit of measurement
☒	☐	A statement on whether measurements were taken from distinct samples or whether the same sample was measured repeatedly
☐	☒	The statistical test(s) used AND whether they are one- or two-sided<br>*Only common tests should be described solely by name; describe more complex techniques in the Methods section.*
☒	☐	A description of all covariates tested
☐	☒	A description of any assumptions or corrections, such as tests of normality and adjustment for multiple comparisons
☐	☒	A full description of the statistical parameters including central tendency (e.g. means) or other basic estimates (e.g. regression coefficient) AND variation (e.g. standard deviation) or associated estimates of uncertainty (e.g. confidence intervals)
☐	☒	For null hypothesis testing, the test statistic (e.g. F, t, r) with confidence intervals, effect sizes, degrees of freedom and P value noted<br>*Give P values as exact values whenever suitable.*
☒	☐	For Bayesian analysis, information on the choice of priors and Markov chain Monte Carlo settings
☒	☐	For hierarchical and complex designs, identification of the appropriate level for tests and full reporting of outcomes
☒	☐	Estimates of effect sizes (e.g. Cohen's d, Pearson's r), indicating how they were calculated

Our web collection on statistics for biologists contains articles on many of the points above.

Software and code

Policy information about availability of computer code

Data collection	We didn't use any software to collect data in this study.
Data analysis	All data passed the normality test using Prism8 software (GraphPad software v8.0; Prism, La Jolla, CA, USA). All flow cytometry data were analyzed by Flowjo X (FlowJo, LLC; Ashland, OR, USA). All western blot blots were analyzed by ImageJ 1.52 (National Institutes of Health, USA).

For manuscripts utilizing custom algorithms or software that are central to the research but not yet described in published literature, software must be made available to editors and reviewers. We strongly encourage code deposition in a community repository (e.g. GitHub). See the Nature Research guidelines for submitting code & software for further information.

Data

Policy information about availability of data

All manuscripts must include a data availability statement. This statement should provide the following information, where applicable:

- Accession codes, unique identifiers, or web links for publicly available datasets
- A list of figures that have associated raw data
- A description of any restrictions on data availability

We have included a data availability statement in the manuscript.

Field-specific reporting

Please select the one below that is the best fit for your research. If you are not sure, read the appropriate sections before making your selection.

☒ Life sciences ☐ Behavioural & social sciences ☐ Ecological, evolutionary & environmental sciences

For a reference copy of the document with all sections, see nature.com/documents/nr-reporting-summary-flat.pdf

Life sciences study design

All studies must disclose on these points even when the disclosure is negative.

Sample size	In previous studies, we observed that the effect size δ for glucose tolerance tests and GSIS is approximately 1.5. Using Russ Lenth's power calculator (University of Iowa), at least 5 mice per group are needed for power of >0.99. Sample sizes are described in figure legends.
Data exclusions	No data were excluded in this study.
Replication	All experiments were repeated at least twice with similar results.
Randomization	All samples such as cells or animals were randomly allocated into experimental groups.
Blinding	Glucose tolerance tests, FACS, immunofluorescent data, qPCR data, and GSIS data were analyzed in a blinded way. Western blot analysis was performed without information about the groups.

Reporting for specific materials, systems and methods

We require information from authors about some types of materials, experimental systems and methods used in many studies. Here, indicate whether each material, system or method listed is relevant to your study. If you are not sure if a list item applies to your research, read the appropriate section before selecting a response.

Materials & experimental systems

n/a	Involved in the study	
☐	☒	Antibodies
☐	☒	Eukaryotic cell lines
☒	☐	Palaeontology and archaeology
☐	☒	Animals and other organisms
☐	☒	Human research participants
☒	☐	Clinical data
☒	☐	Dual use research of concern

Methods

n/a	Involved in the study	
☒	☐	ChIP-seq
☐	☒	Flow cytometry
☒	☐	MRI-based neuroimaging

Antibodies

Antibodies used

Anti-CD63 (ABclonal, Cat# A5271; RRID: AB_2766092; WB-1:2000);
Anti-HSP90 (Santa Cruz, Cat# sc-101494; RRID: AB_1124018; WB-1:2000);
Anti-GAPDH (Cell signaling technology, Cat# 2118S; RRID: AB_561053; WB-1:2000);
Anti-HSP70 (ThermoFisher, Cat# MA5-31961; RRID: AB_2809255; WB-1:2000);
APC/Cyanine7 anti-CD45 (BioLegend, Cat# 103115; RRID: AB_312980; FACS-1:200);
FITC anti-CD11b (BioLegend, Cat# 101205; RRID: AB_312788; FACS-1:200);
PE/Cy7 anti-F4/80 (BioLegend, Cat# 123113; RRID: AB_893490; FACS-1:200);
Anti-Vsig4 (ThermoFisher, Cat# 17-5752-82; RRID: AB_2637429; IF-1:100, WB-1:2000);
Anti-mouse cGAS (Cell signaling technology, Cat# 316595; RRID: AB_2799008; WB-1:2000);
Anti-human cGAS (Cell signaling technology, Cat# 15102; RRID: AB_2732795; WB-1:2000);
Anti-mouse STING (Cell signaling technology, Cat# 50494; RRID: AB_2799375; WB-1:2000);
Anti-human STING (Cell signaling technology, Cat# 13647; RRID: AB_2732796; WB-1:2000);
Anti-mouse pSTING (Cell signaling technology, Cat# 72971; RRID: AB_2799831; WB-1:2000);
Anti-human pSTING (Cell signaling technology, Cat# 50907; RRID: AB_2827656; WB-1:2000)
Anti-CD115 (10μg/10-30 islets; Cat# BE0213, BioXCell)

Validation

Anti-CD63 (ABclonal, Cat# A5271; reactivity: human, mouse, rat; citations provided by the manufacturer: https://abclonal.com/catalog-antibodies/CD63RabbitpAb/A5271);
Anti-HSP90 (Santa Cruz, Cat# sc-101494; reactivity: human, mouse, rat; citations provided by the manufacturer: https://www.scbt.com/p/hsp-90-antibody-ac-16);
Anti-GAPDH (Cell signaling technology, Cat# 2118S; reactivity: human, mouse, rat, monkey, pig; citations provided by the manufacturer: https://www.cellsignal.com/products/primary-antibodies/gapdh-14c10-rabbit-mab/2118);
Anti-HSP70 (ThermoFisher, Cat# MA5-31961; reactivity: human, mouse, rat; citations provided by the manufacturer: https://

www.thermofisher.com/antibody/product/HSP70-Antibody-clone-SA0379-Recombinant-Monoclonal/MA5-31961);
APC/Cyanine7 anti-CD45 (BioLegend, Cat# 103115; reactivity: mouse; citations provided by the manufacturer: https://www.biolegend.com/en-us/products/apc-cyanine7-anti-mouse-cd45-antibody-2530?GroupID=BLG1932);
FITC anti-CD11b (BioLegend, Cat# 101205; reactivity: mouse, human; citations provided by the manufacturer: https://www.biolegend.com/en-us/products/fitc-anti-mouse-human-cd11b-antibody-347?GroupID=BLG10660);
PE/Cy7 anti-F4/80 (BioLegend, Cat# 123113; reactivity: mouse; citations provided by the manufacturer: https://www.biolegend.com/en-us/products/pe-cyanine7-anti-mouse-f4-80-antibody-4070?GroupID=BLG5319);
Anti-Vsig4 (ThermoFisher, Cat# 17-5752-82; reactivity: mouse, human; citations provided by the manufacturer: https://www.thermofisher.com/antibody/product/VSIG4-Antibody-clone-NLA14-Monoclonal/17-5752-82);
Anti-mouse cGAS (Cell signaling technology, Cat# 316595; reactivity: mouse; citations provided by the manufacturer: https://www.cellsignal.com/products/primary-antibodies/cgas-d3o8o-rabbit-mab-mouse-specific/31659);
Anti-human cGAS (Cell signaling technology, Cat# 15102; reactivity: human; citations provided by the manufacturer: https://www.cellsignal.com/products/primary-antibodies/cgas-d1d3g-rabbit-mab/15102);
Anti-mouse STING (Cell signaling technology, Cat# 50494; reactivity: mouse, human, rat; citations provided by the manufacturer: https://www.cellsignal.com/products/primary-antibodies/sting-d1v5l-rabbit-mab-rodent-preferred/50494);
Anti-human STING (Cell signaling technology, Cat# 13647; reactivity: mouse, human; citations provided by the manufacturer: https://www.cellsignal.com/products/primary-antibodies/sting-d2p2f-rabbit-mab/13647);
Anti-mouse pSTING (Cell signaling technology, Cat# 72971; reactivity: mouse; citations provided by the manufacturer: https://www.cellsignal.com/products/primary-antibodies/phospho-sting-ser365-d8f4w-rabbit-mab/72971);
Anti-human pSTING (Cell signaling technology, Cat# 50907; reactivity: human; citations provided by the manufacturer: https://www.cellsignal.com/products/primary-antibodies/phospho-sting-ser366-e9a9k-rabbit-mab/50907?site-search-type=Products&N=4294956287&Ntt=50907&fromPage=plp&_requestid=155247)
Anti-CD115 (Cat# BE0213, BioXCell; reactivity: mouse; citations provided by the manufacturer: https://bxcell.com/product/anti-cd115-anti-csf-1/)

Eukaryotic cell lines

Policy information about cell lines

Cell line source(s)	Min6 cells were received from AddexBio.
Authentication	The authentication of these cell lines have been confirmed by AddexBio (morphological criteria and glucose-stimulated insulin secretion. etc).
Mycoplasma contamination	Mycoplasma contamination tests were done when culturing cells. All tests were mycoplasma negative.
Commonly misidentified lines (See ICLAC register)	We didn't use any misidentified cell lines in this study.

Animals and other organisms

Policy information about studies involving animals; ARRIVE guidelines recommended for reporting animal research

Laboratory animals	All mice used in this paper were male and started special diet feeding from 8 weeks old. WT C57BL6/J (JAX, Stock No. 000664), cGAS knockout (JAX, Stock No. 026554), C3 knockout (JAX, Stock No. 029661), ROSA26iDTR (JAX, Stock No. 007900), Clec4f Cre (JAX, Stock No. 033296), MIPGFP (JAX, Stock No. 006864), Ins Cre (JAX, Stock No. 026801), Sting flox mice (JAX, Stock No. 031670), Vsig4 knockout (maintained by Dr. Wei Ying's lab in UCSD)
Wild animals	We didn't use any wild animals in this study.
Field-collected samples	This study did not use any field-collected samples
Ethics oversight	All animal procedures complied with all relevant ethical regulations and were conducted under approved protocols (Protocol# S19147) by University of California, San Diego Research Guidelines for the Care and Use of Laboratory Animals

Note that full information on the approval of the study protocol must also be provided in the manuscript.

Human research participants

Policy information about studies involving human research participants

Population characteristics	All human samples used in this paper were derived from lean/healthy individuals or patients with obesity/type 2 diabetes. The detailed information is provided in Supplementary table 1 and 2.
Recruitment	N/A
Ethics oversight	The UCSD IRB has waived the review and approval for the usage of human islets (Prodo Laboratories; purity >90%; Islet purity was determined by staining with Diphenylthiocarbozone to distinguish islets from non-islet tissue.) or pancreatic samples (Novus Biologicals or International institute for the advancement of medicine) (UCSD IRB exempt protocol# 801547) due to the secondary usage of existing human pancreatic biospecimens. The information related to human islets used in this study is provided in Supplementary table 1.<br>Archival human plasma samples used in this study were from generally healthy, overnight-fasted lean and obese, non-diabetic men residing in San Diego County. Samples were collected in accordance with the Declaration of Helsinki and the

principles of Good Clinical Practice as part of the Community of Mine Study, approved by the UC San Diego Institutional Review Board (Jankowska et al., BMC Public Health, 2019). All participants provided written informed consent.

Note that full information on the approval of the study protocol must also be provided in the manuscript.

Flow Cytometry

Plots

Confirm that:

- [x] The axis labels state the marker and fluorochrome used (e.g. CD4-FITC).
- [x] The axis scales are clearly visible. Include numbers along axes only for bottom left plot of group (a 'group' is an analysis of identical markers).
- [x] All plots are contour plots with outliers or pseudocolor plots.
- [x] A numerical value for number of cells or percentage (with statistics) is provided.

Methodology

Sample preparation	Liver nonparechymal cells were isolated and stained with antibodies (CD11b, F4/80, CD45) for measuring macrophages (CD11b+F4/80+). Islet single cells were stained with antibodies (CD11b, F4/80, CD45) for measuring macrophages (CD11b +F4/80+).
Instrument	SONY MA900
Software	FlowJo
Cell population abundance	Macrophage population is usually about 30-40% among total CD45+ cells in liver or about 15% of viable islet cells.
Gating strategy	We first gated single cell population using FSH/FSA, and then excluded dead cells using aqua live/dead staining. The population of macrophages (CD11b+F4/80+) were analyzed as described in our previous papers (Ying et al. 2019, Cell metabolism; Luo et al. 2021, Gatroenterology)

- [x] Tick this box to confirm that a figure exemplifying the gating strategy is provided in the Supplementary Information.
