# Supplementary figures and images for: Accumulation of microbial DNAs promotes to islet inflammation and β cell abnormalities in obesity in mice
Source: Nat Commun. 2022 Jan 28;13:565. doi: 10.1038/s41467-022-28239-2 (PMC8799656; doi:10.1038/s41467-022-28239-2)

Figure S1f

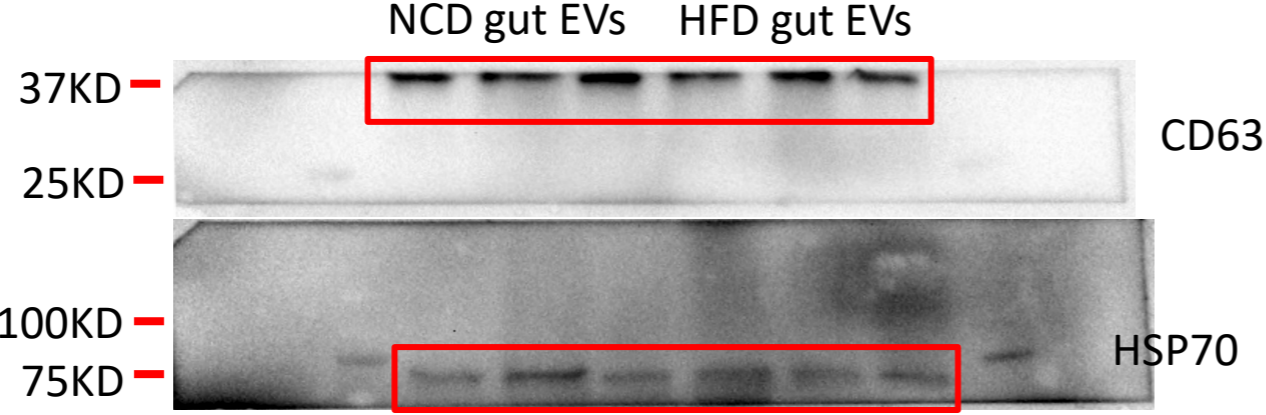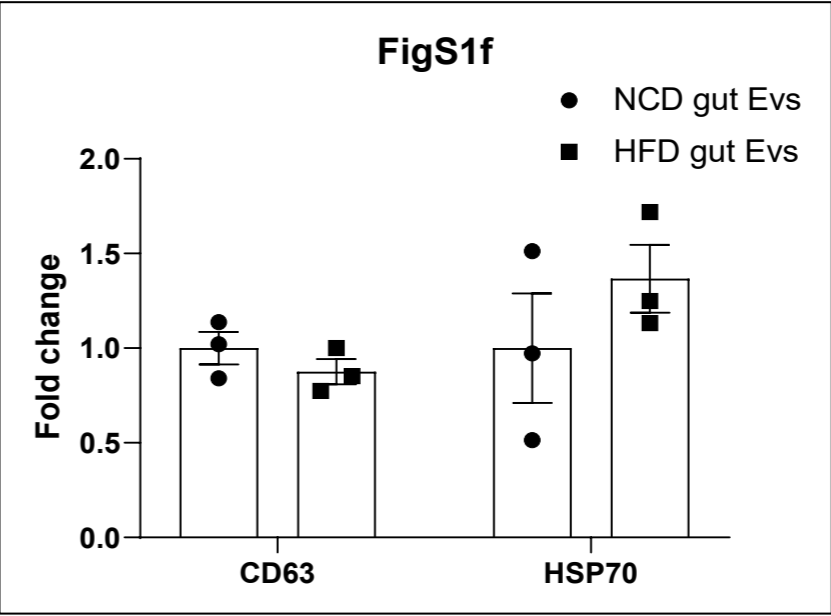

**Figure 2f**

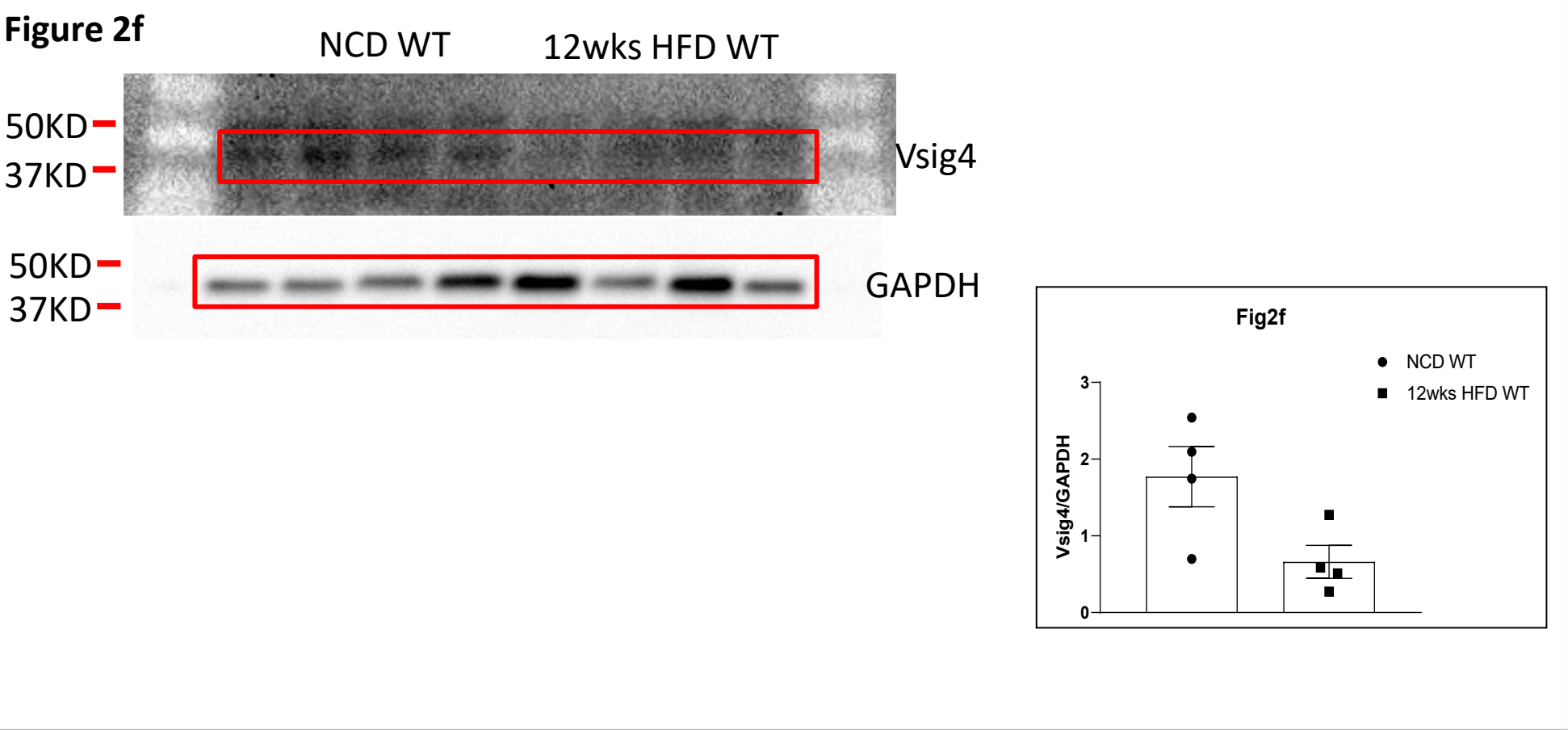

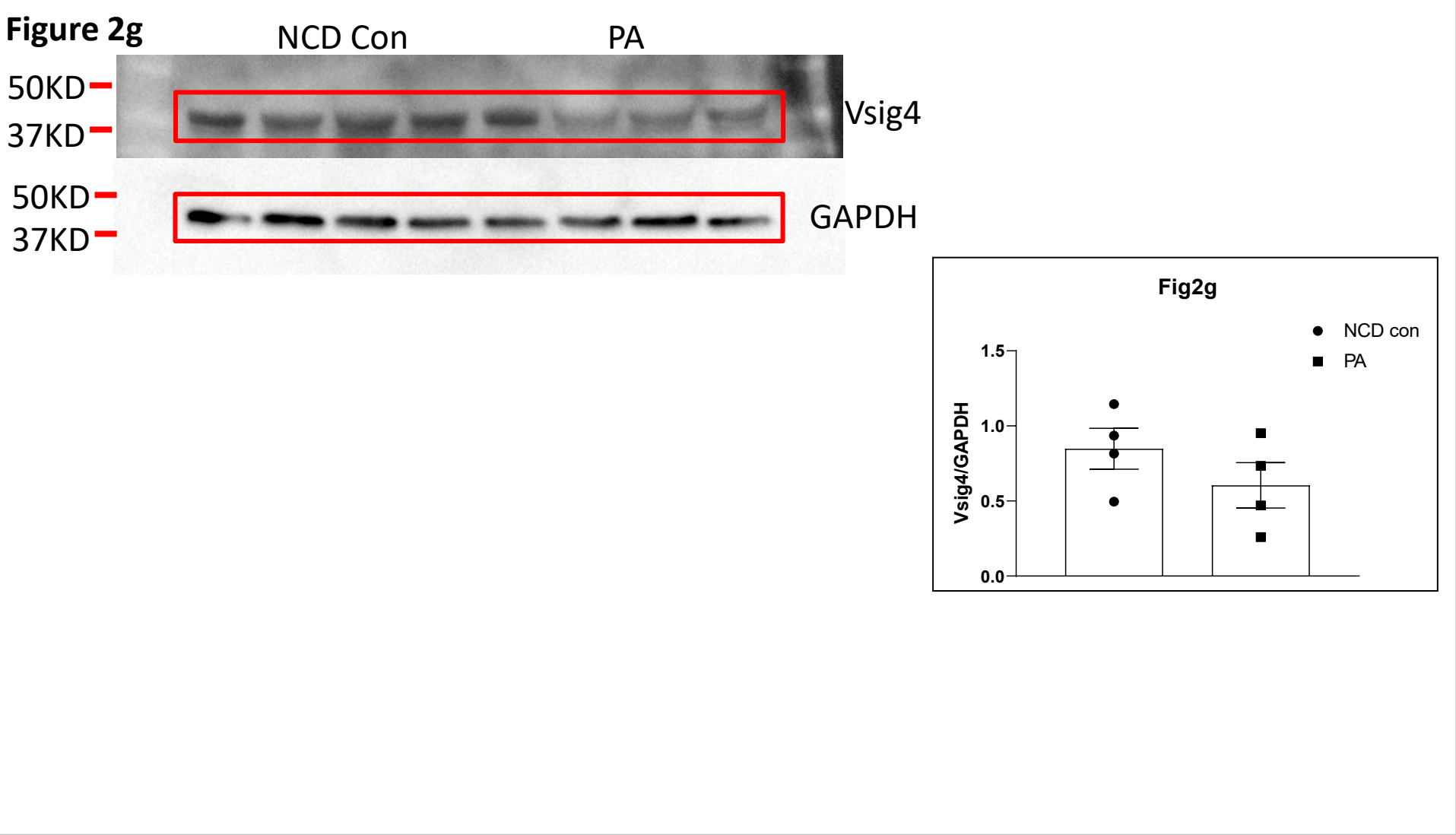

**Figure S2f**

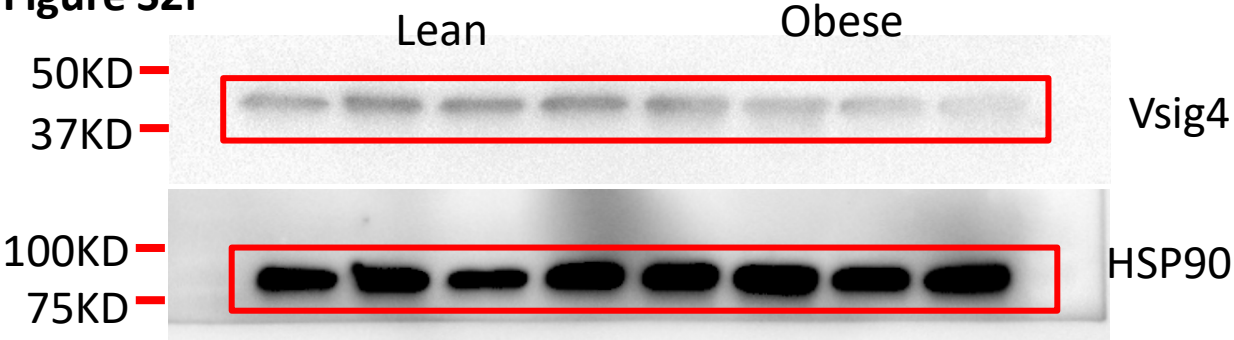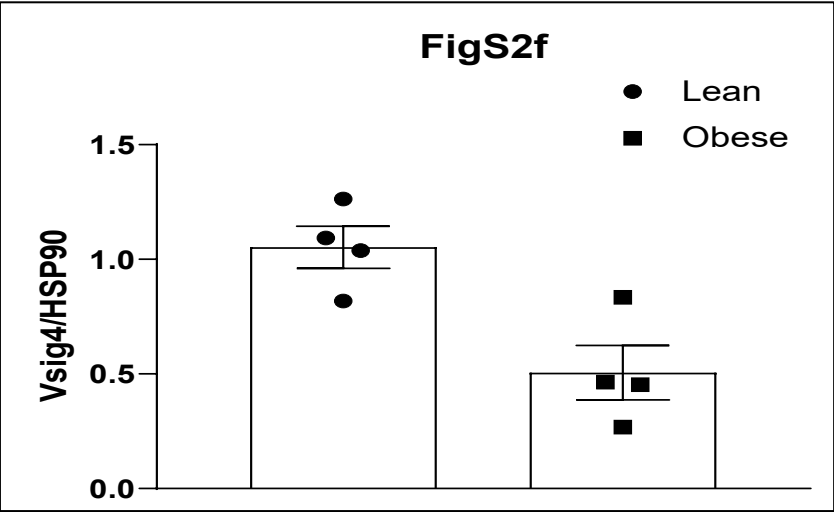

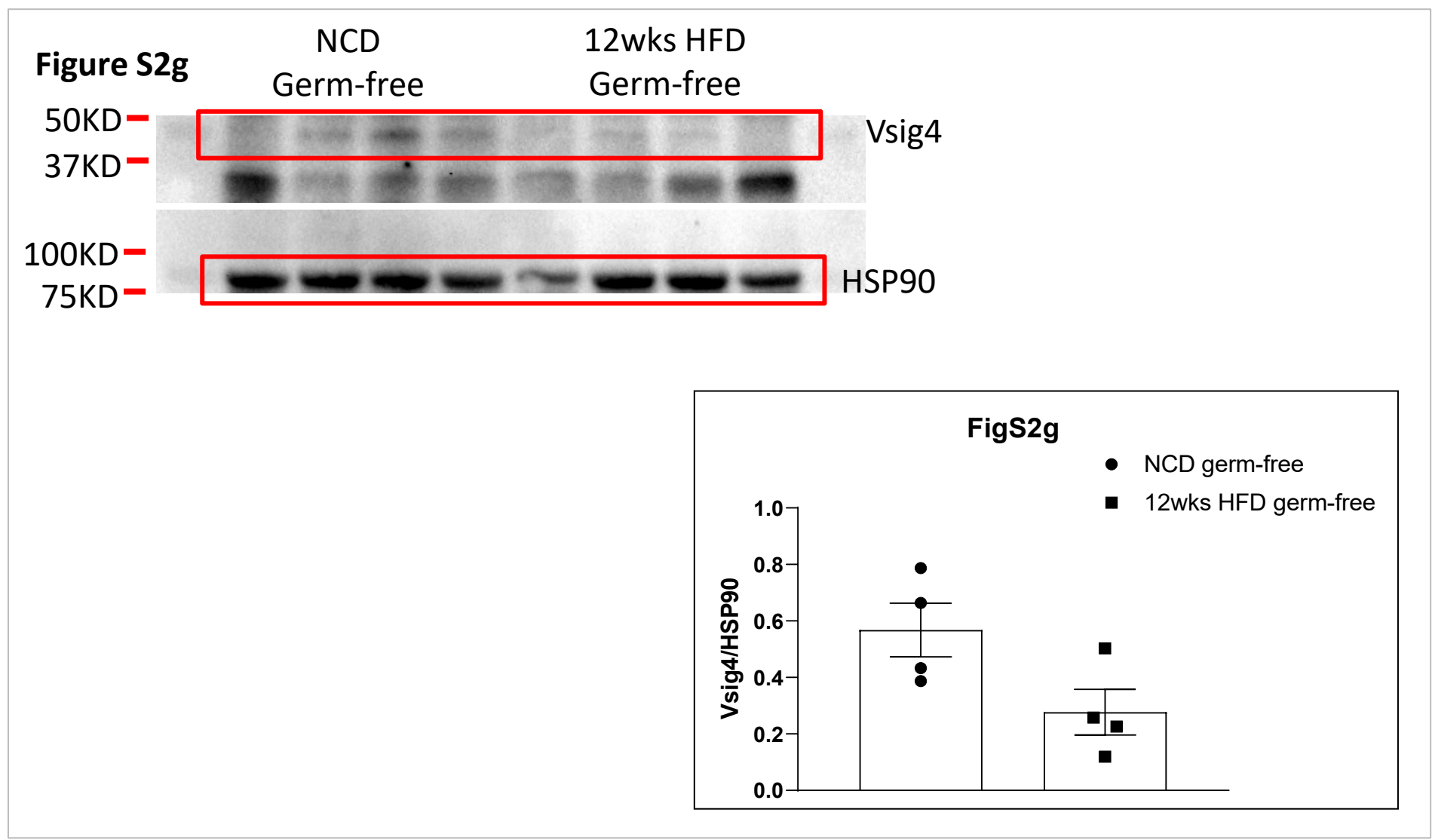

Figure S5b

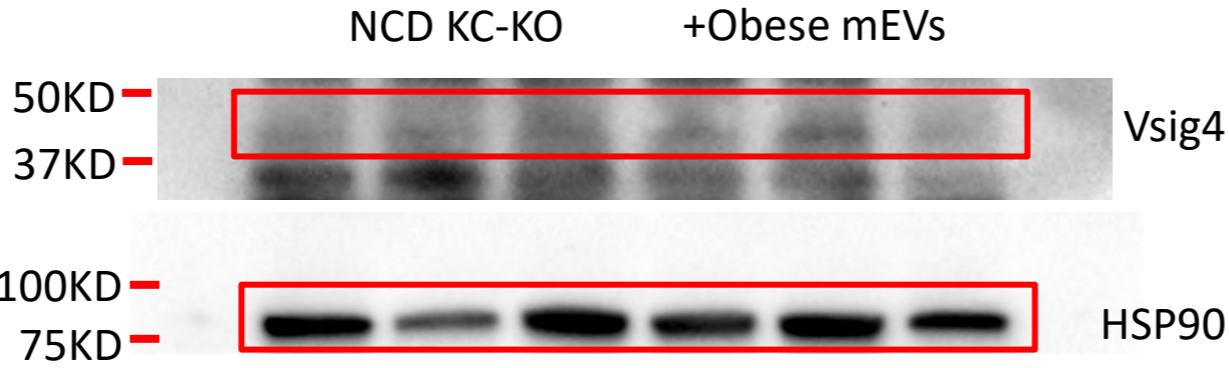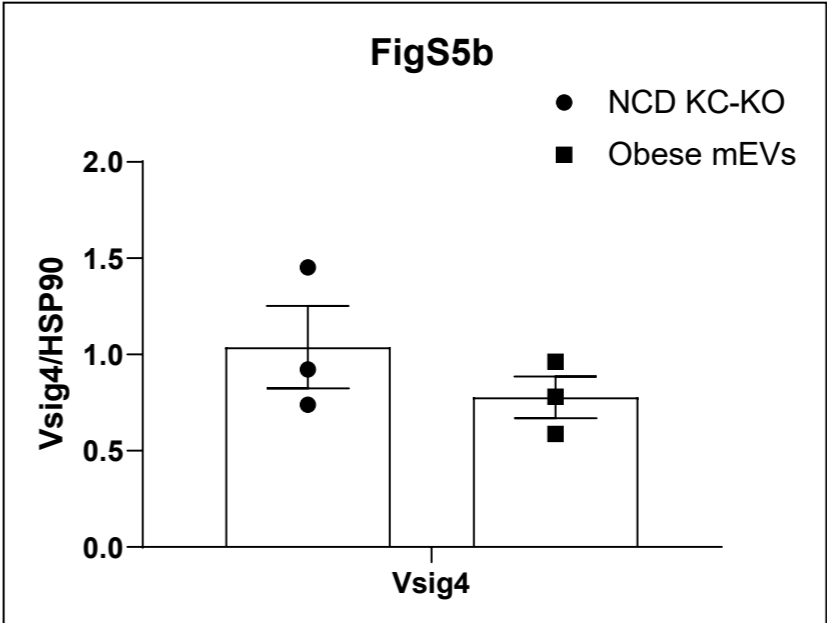

**Figure S5d**

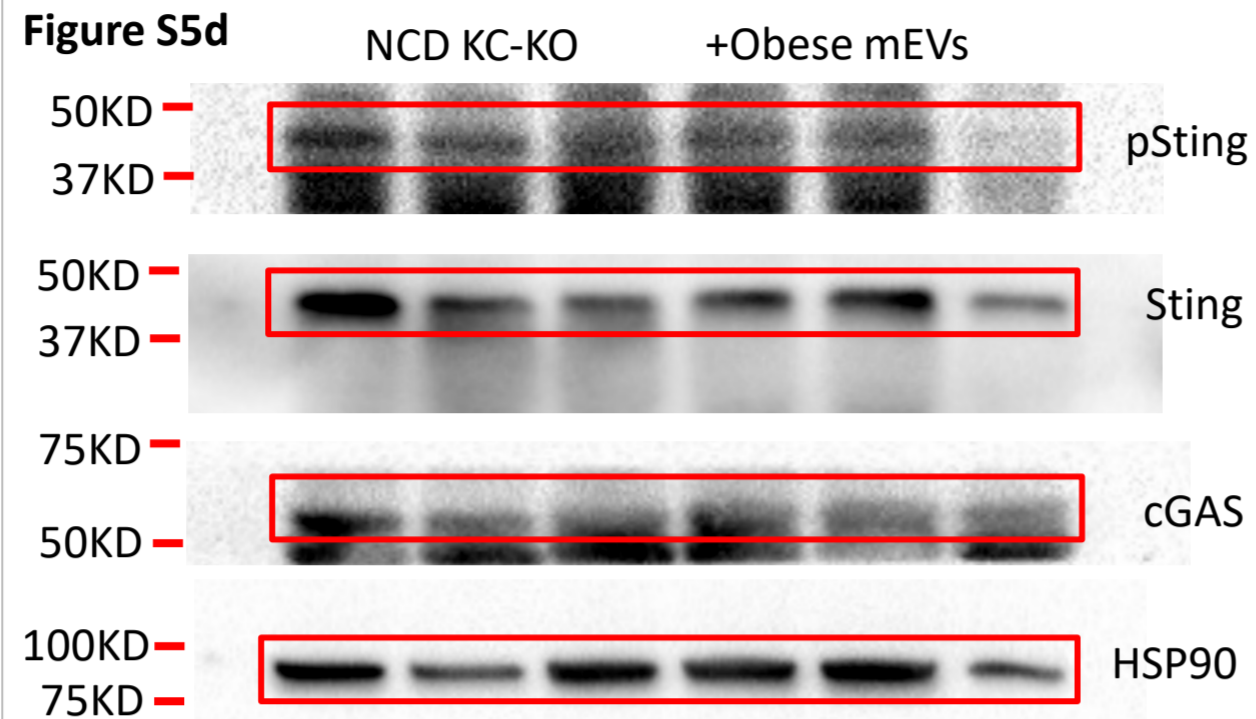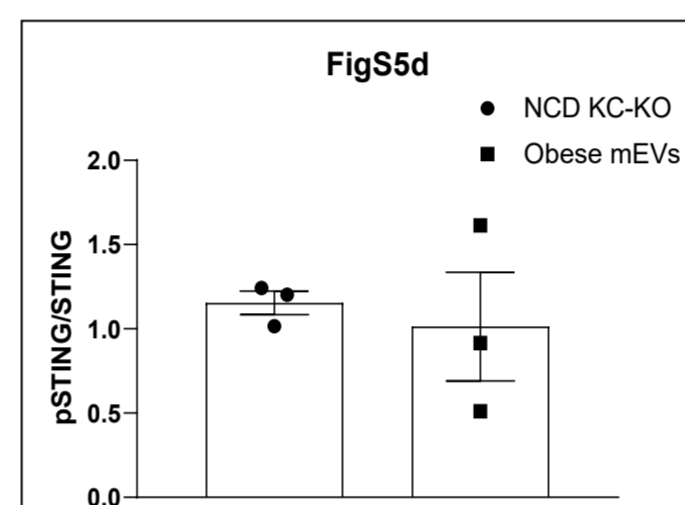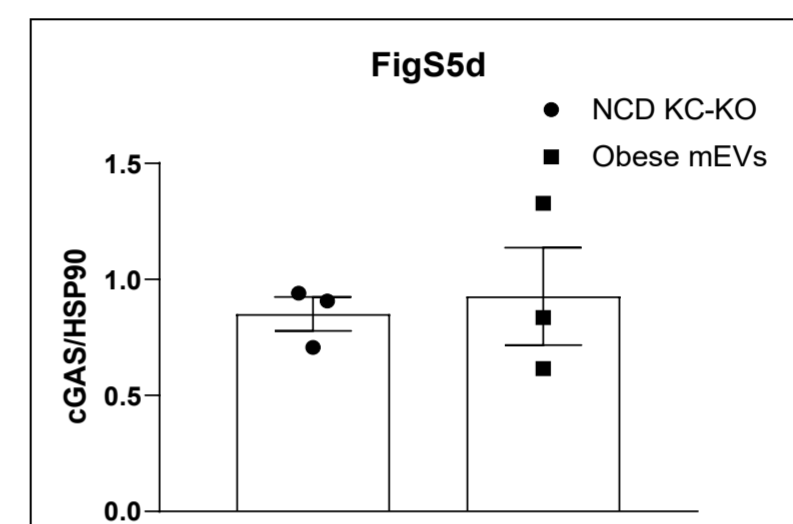

**Figure 7a**

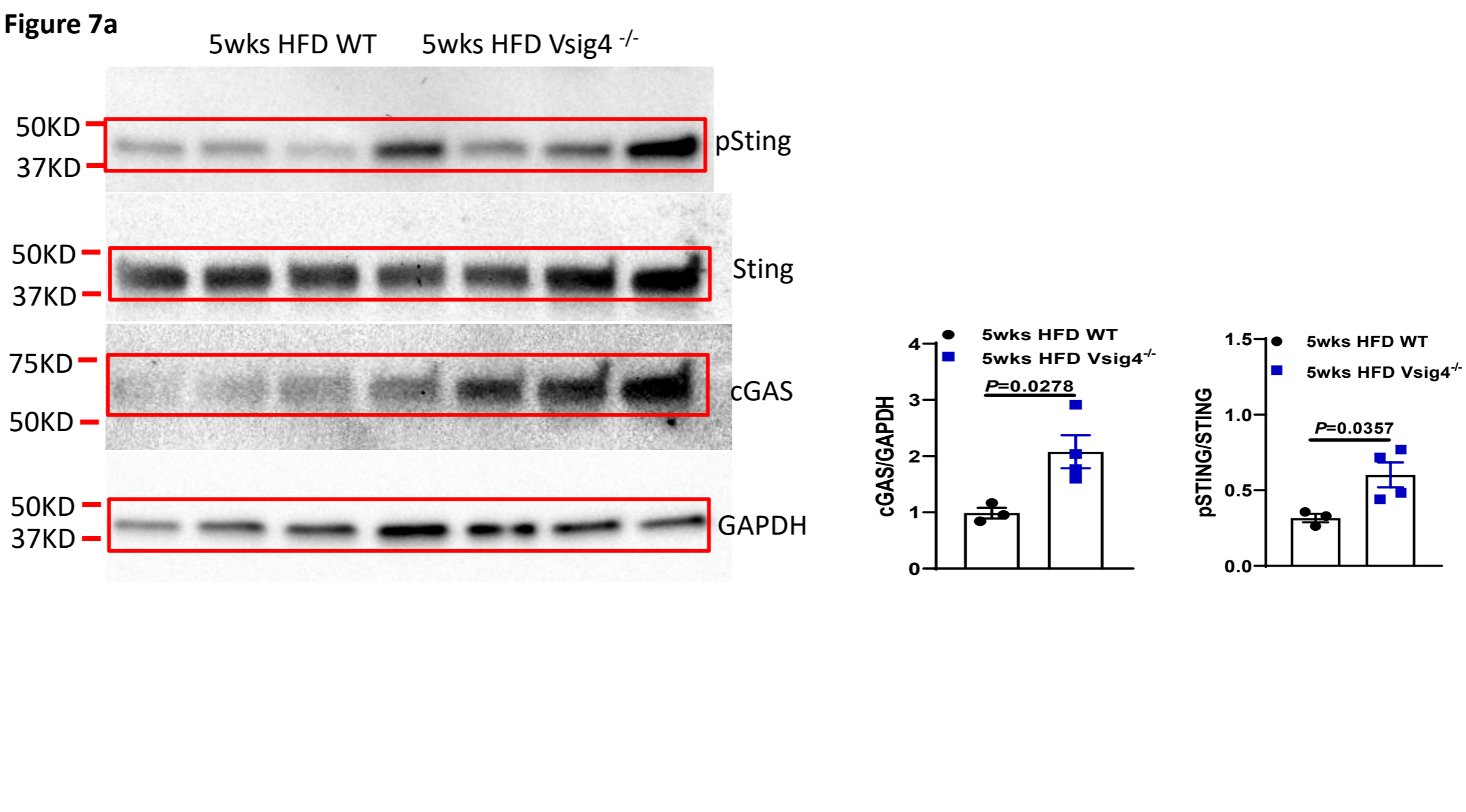

**Figure 7b**

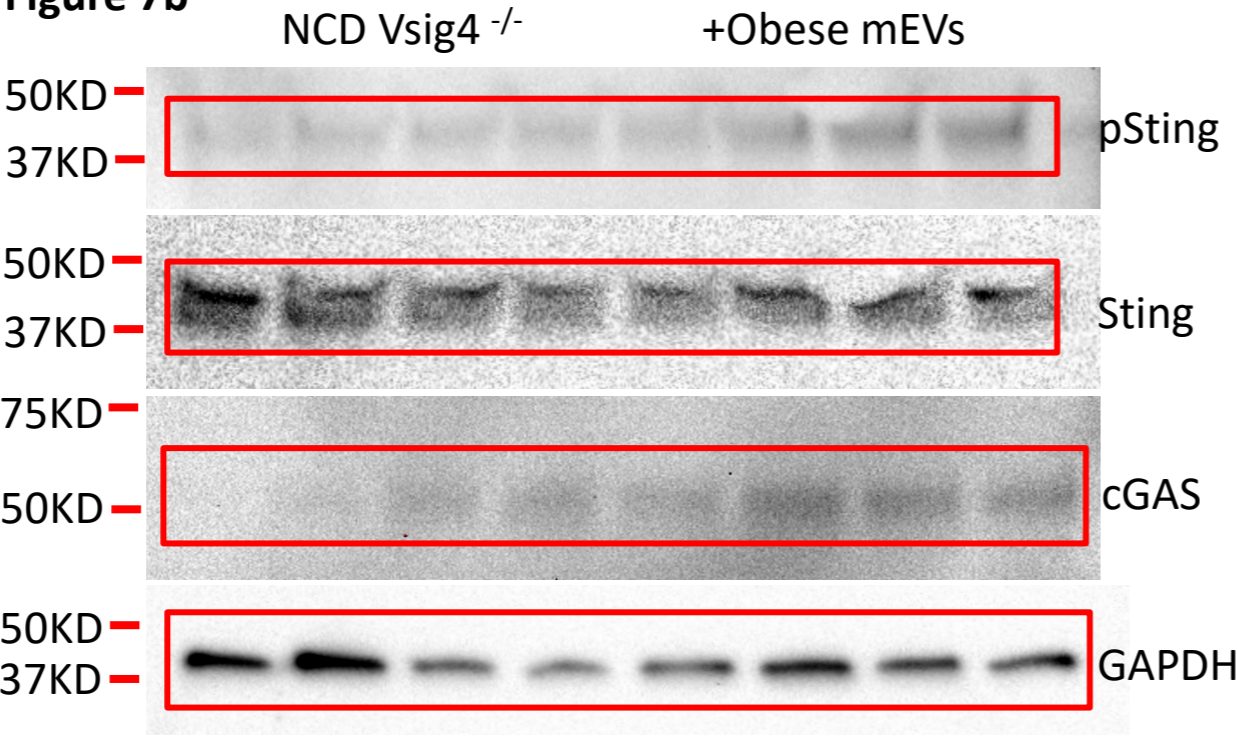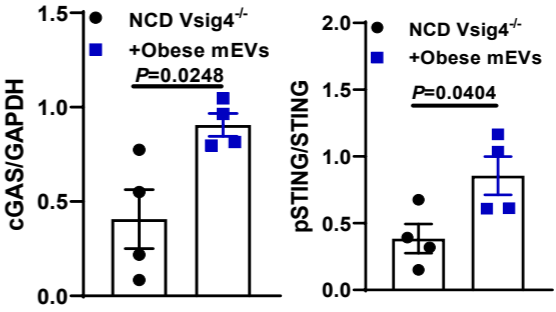

**Figure 7c**

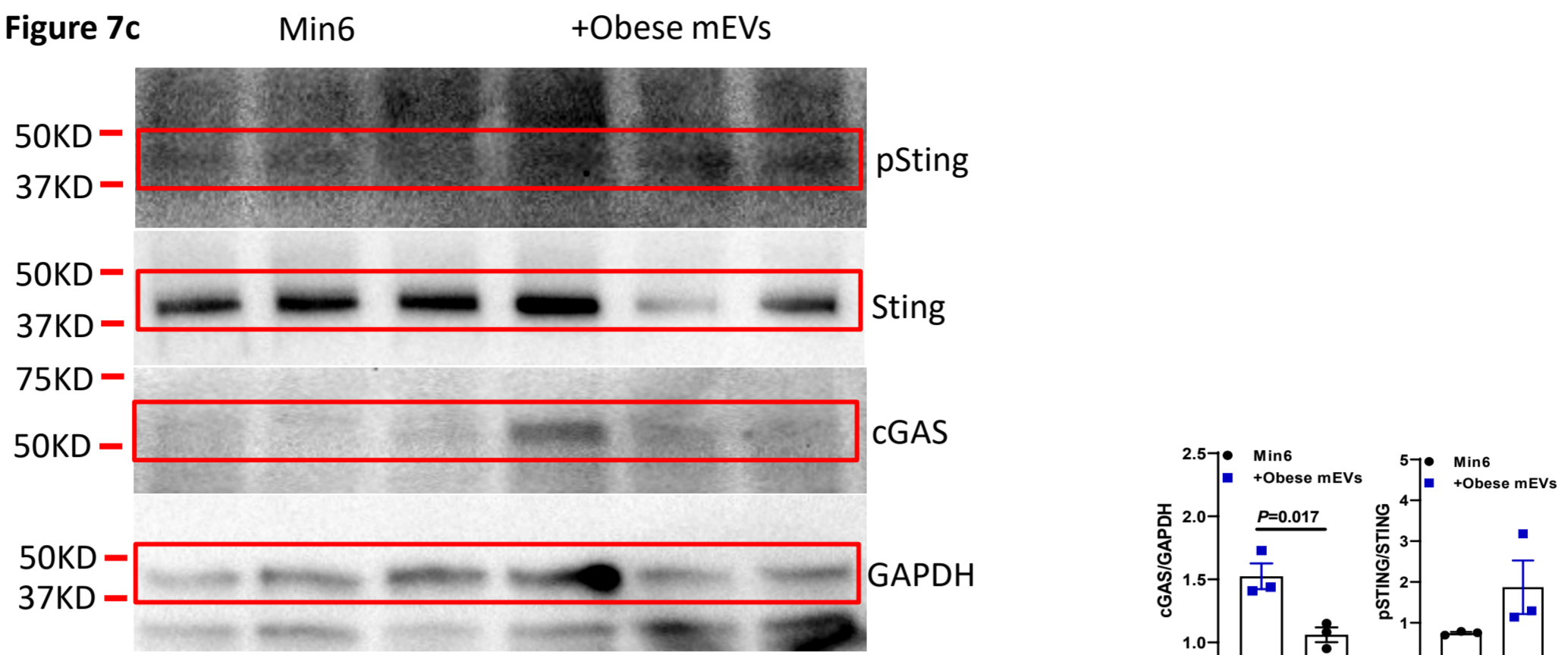

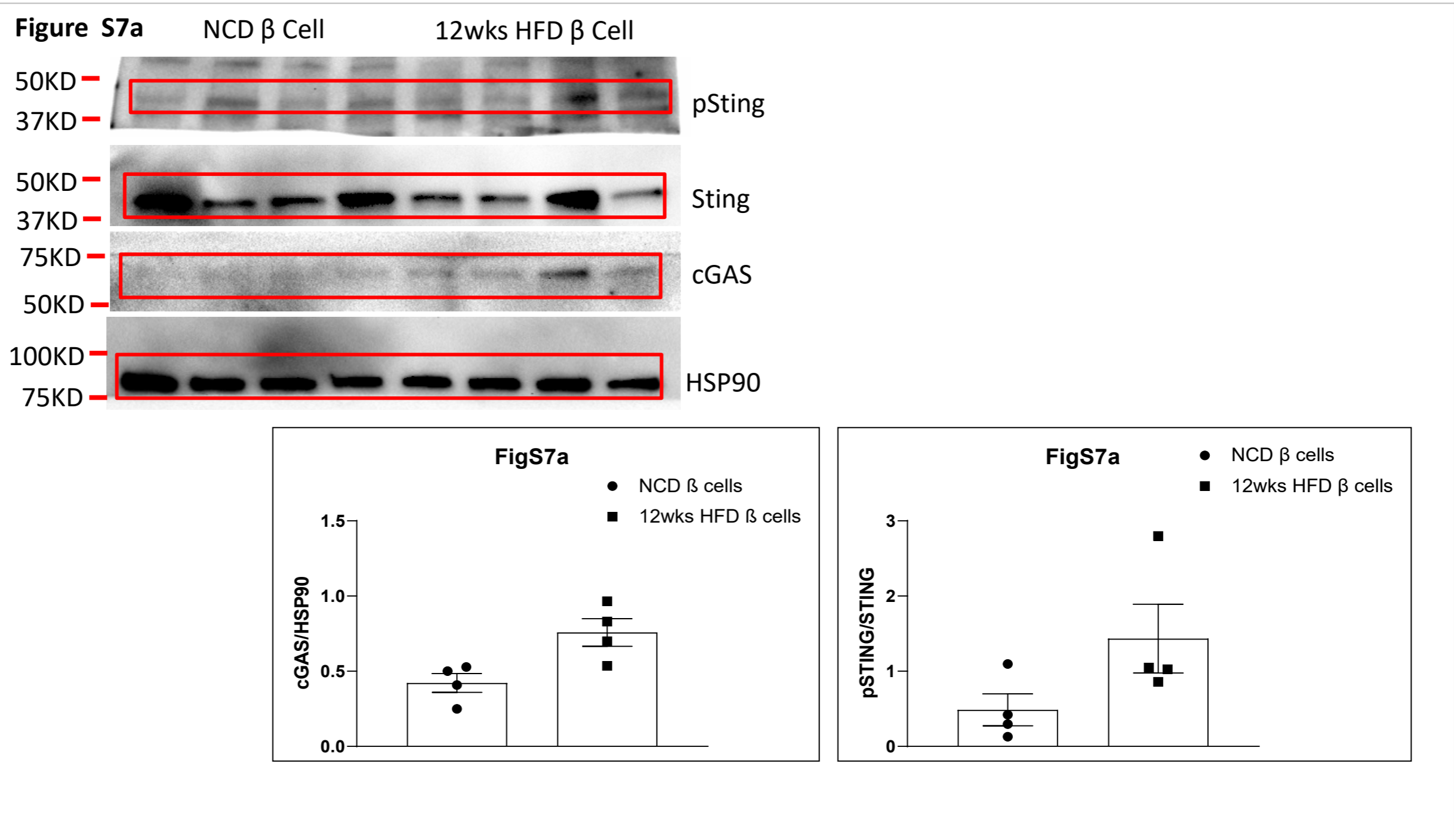

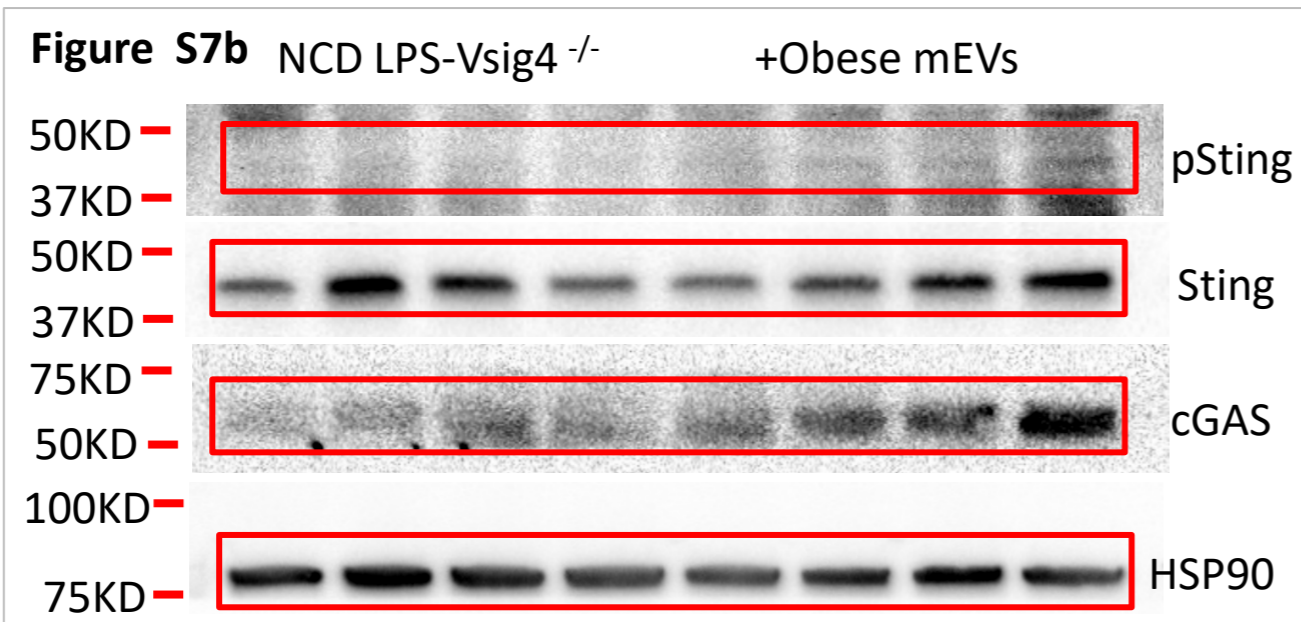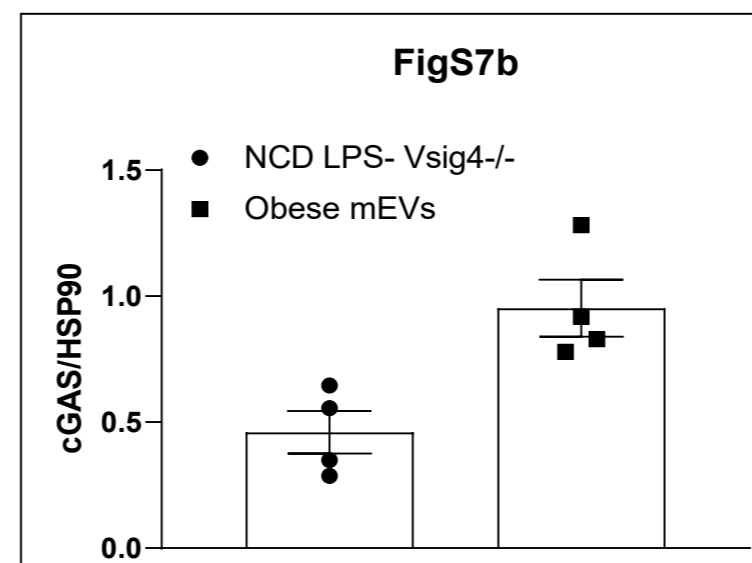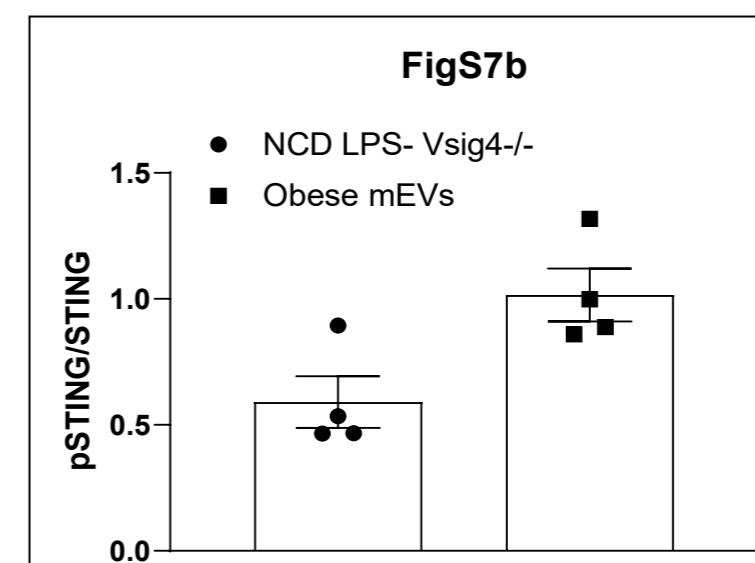

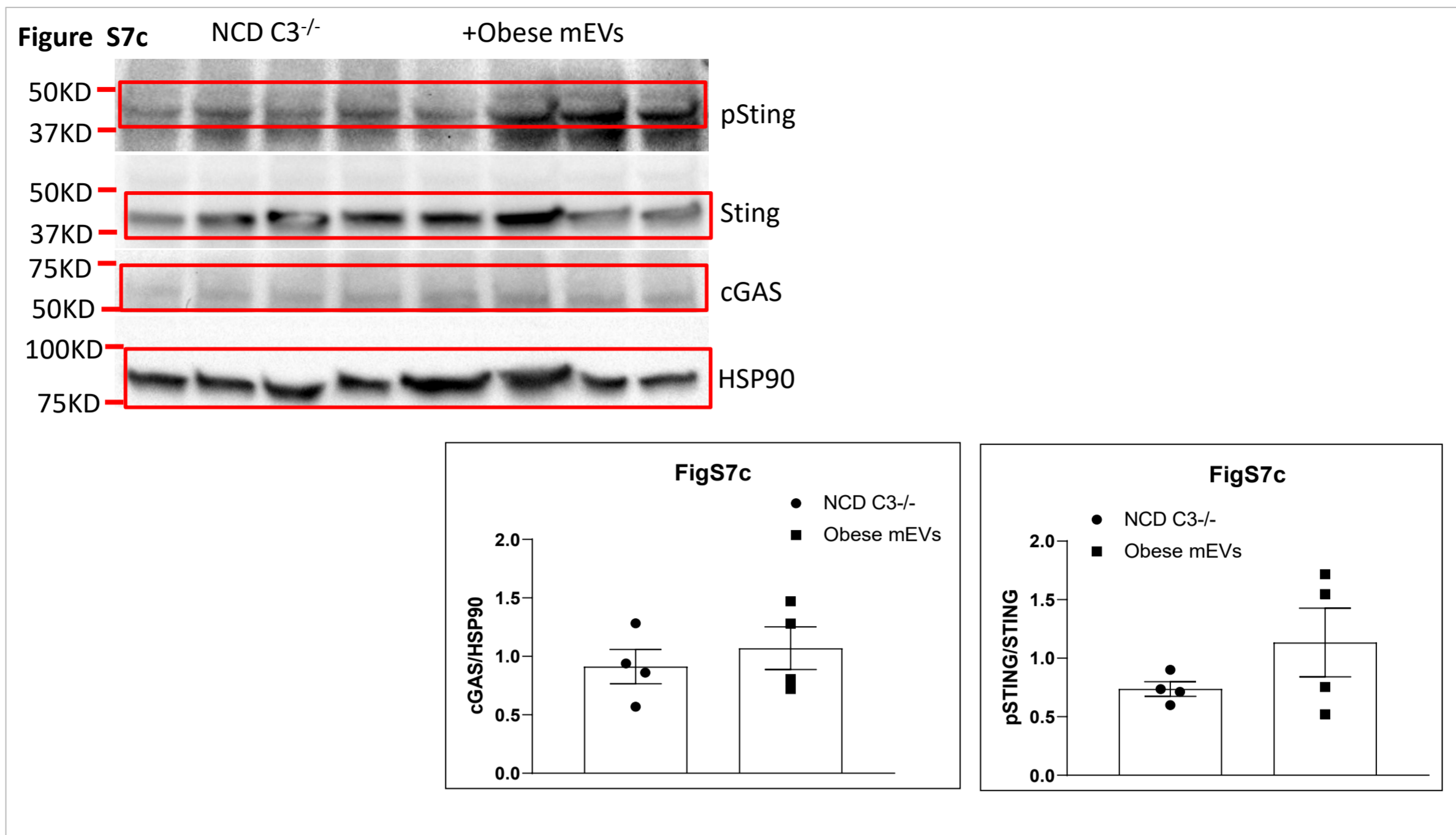

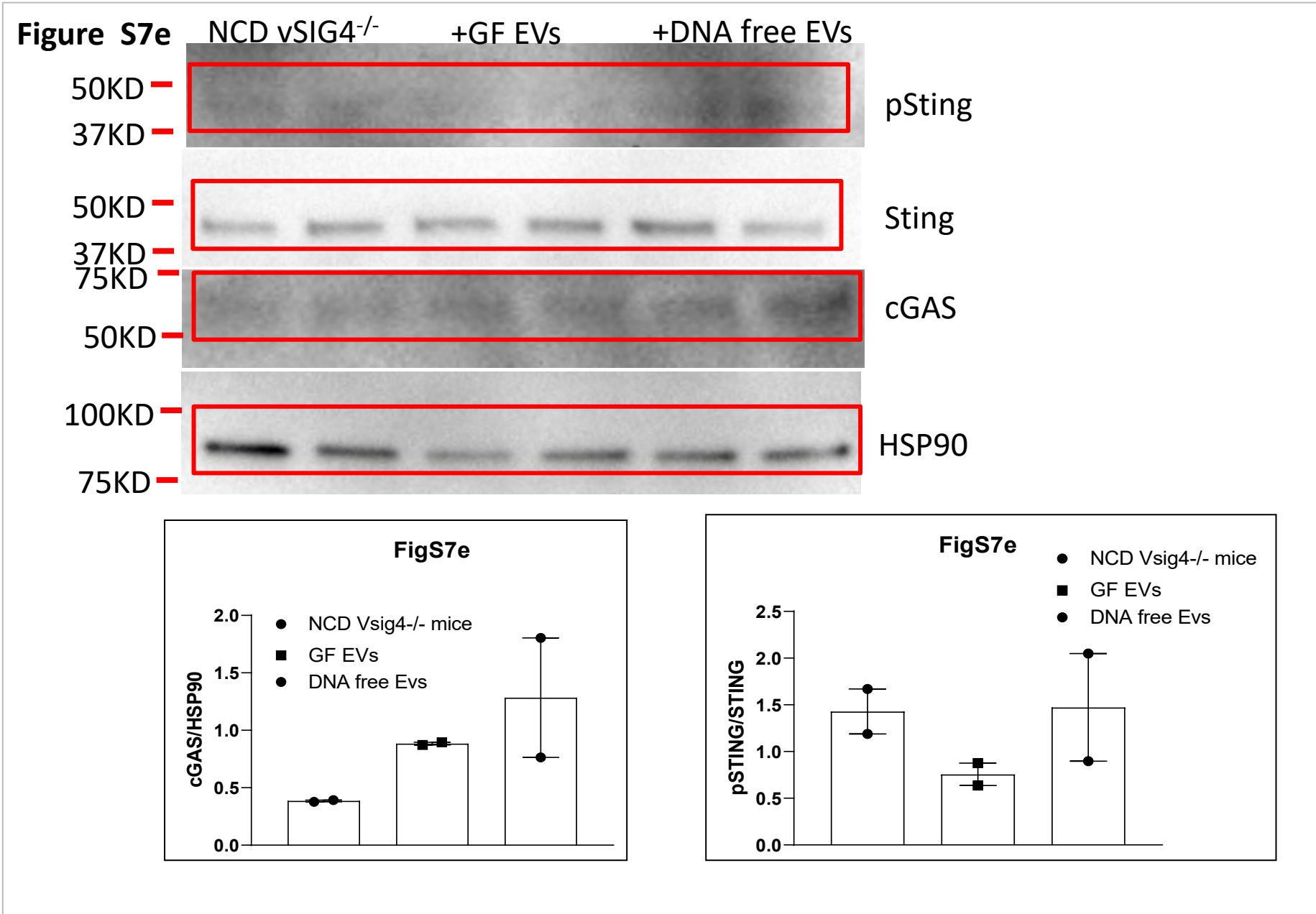

Supplement: Supplementary file 3 — Source Data [file 41467_2022_28239_MOESM3_ESM.pdf]
